# Wide diversity in narrow geographic space: genetic, morphological and ploidy variation in three Central European *Crataegus* species with emphasis on their reproductive modes

**DOI:** 10.1093/aobpla/plaf067

**Published:** 2025-11-29

**Authors:** Soňa Píšová, Roman Ufimov, Michaela Breuer, Saranchimeg Jargal, Lok Sze Florence Lee, Anna Zöchner, Paula Irusta, Eliah Milan Grooß, Tomáš Fér, Roswitha Schmickl, Marcela van Loo

**Affiliations:** Department of Forest Biodiversity and Nature Conservation, Austrian Research Centre for Forests, Seckendorff-Gudent-Weg 8, 1131 Vienna, Austria; Department of Forest Growth, Silviculture, and Genetics, Austrian Research Centre for Forests, Seckendorff-Gudent-Weg 8, 1131 Vienna, Austria; Russian Academy of Sciences, Komarov Botanical Institute, Ulitsa Professora Popova 2, 197022 St. Petersburg, Russian Federation; Department of Forest Growth, Silviculture, and Genetics, Austrian Research Centre for Forests, Seckendorff-Gudent-Weg 8, 1131 Vienna, Austria; Department of Forest Growth, Silviculture, and Genetics, Austrian Research Centre for Forests, Seckendorff-Gudent-Weg 8, 1131 Vienna, Austria; Faculty of Life Sciences, University of Vienna, Rennweg 14, 1030 Vienna, Austria; Department of Forest Growth, Silviculture, and Genetics, Austrian Research Centre for Forests, Seckendorff-Gudent-Weg 8, 1131 Vienna, Austria; Department of Silviculture, BOKU University, Peter-Jordan-Straße 82/2, 1190 Vienna, Austria; Department of Forest Growth, Silviculture, and Genetics, Austrian Research Centre for Forests, Seckendorff-Gudent-Weg 8, 1131 Vienna, Austria; Department of Biomedical Imaging and Image-Guided Therapy, Medical University of Vienna, Währinger Gürtel 18-20, 1090 Vienna, Austria; Department of Forest Growth, Silviculture, and Genetics, Austrian Research Centre for Forests, Seckendorff-Gudent-Weg 8, 1131 Vienna, Austria; Faculty of Biology, University of Barcelona, Diagonal 643, 08028 Barcelona, Spain; Faculty of Life Science, Leipzig University, Talstraße 33, 04103 Leipzig, Germany; Department of Conservation Biology, University of Göttingen, Bürgerstrasse 50, 37073 Göttingen, Germany; Department of Botany, Faculty of Science, Charles University, Benátská 2, 128 00 Prague, Czech Republic; Department of Botany, Faculty of Science, Charles University, Benátská 2, 128 00 Prague, Czech Republic; Institute of Botany, Czech Academy of Sciences, Zámek 1, 252 43 Průhonice, Czech Republic; Department of Forest Growth, Silviculture, and Genetics, Austrian Research Centre for Forests, Seckendorff-Gudent-Weg 8, 1131 Vienna, Austria; Molecular Function & Environment

**Keywords:** hybridization, reticulate evolution, ploidy levels, apomixis, reproductive modes, *Crataegus*

## Abstract

Hybridization, polyploidization, and apomixis are evolutionary forces that obscure genetic differentiation and boost morphological variability. These processes have shaped the family Rosaceae, particularly the genus *Crataegus*, which includes both diploid and polyploid species reproducing sexually or via apomixis. In Central Europe, *C. monogyna* and *C. laevigata* are predominantly diploid sexuals, while *C. rhipidophylla* is mainly a polyploid apomict. These species hybridize to form *C.*  *×*  *media*, *C.*  *×*  *macrocarpa*, and *C.*  *×*  *subsphaerica*. Our aim was to assess how hybridization, apomixis, and polyploidy shape *Crataegus* diversity by integrating genetic, morphological, and cytological data. Leaves and fruits were collected from ten natural populations where all three species coexist and hybridize. Species identification was performed with novel nuclear microsatellites, marking the first genetic-based *Crataegus* taxonomy in Central Europe. Ploidy levels were estimated by flow cytometry (FCM), including seed screening to infer reproductive modes. A combined morphological analysis of leaves and fruits was used to distinguish parental species and evaluate hybrid variability. Genotyping identified distinct genetic clusters for parental species and their hybrids, with geographic structuring within *C. laevigata* and *C. rhipidophylla*. Morphological data clearly separated genetically defined parental species, although hybrids can be difficult to distinguish from parents due to a big overlap in morphology. FCM indicated that *C.*  *×*  *media* is predominantly a diploid sexual hybrid like its diploid parents, while other tri- or tetraploid hybrids with polyploid *C. rhipidophylla* as a parent are apomictic. Ploidy rather than hybridization dictates the mode of reproduction.

## Teaser

Hybridization, polyploidy, and asexual seed formation can blur species boundaries in plants and create complex patterns of variation. To understand how these processes interact in natural populations, we studied three Central European hawthorn species and their hybrids using newly developed genetic markers, leaf and fruit morphology, and flow cytometry of both plants and seeds. We show that species remain genetically distinct despite frequent hybridization, and that reproductive mode is largely driven by ploidy rather than hybrid origin: diploids reproduce sexually, while polyploids and their hybrids are mostly apomictic. These contrasting reproductive strategies help explain patterns of clonality, gene flow, and morphological overlap. Our work highlights the value of integrating genetic, cytological, and morphological data to resolve complex plant groups.

## Introduction

Apomictic plant groups reproduce asexually via seeds, resulting in clonal maternal offspring. As such, they are a fascinating target to study the evolution of mating system differences in relation to hybridization and polyploidy (Hojsgaard and Hörandl 2019, Hörandl et al. 2024). In addition, they also constitute a taxonomic nightmare, which calls for novel approaches to integrative taxonomy (Karbstein et al. 2024). Apomictic taxa tend to be of hybrid origin (Paun et al. 2006, Beck et al. 2012, Šarhanová et al. 2017), and are common in major families such as Asteraceae, Poaceae, Ranunculaceae, and Rosaceae (Hörandl et al. 2024). Nevertheless, it appears that in addition to being hybrids, the vast majority of apomictic plants are also polyploid, i.e. they possess multiple chromosome sets. Intriguingly, apomixis is sometimes expressed without any signs of hybridity or polyploidy, such as in the genera *Böchera* Á.Löve & D.Löve (Brassicaceae) (Böcher 1951) and *Paspalum* L. (Poaceae) (Ortiz et al. 2013). Environmental conditions may as well affect the expression of apomixis (Klatt et al. 2018).

Within the Rosaceae, polyploidy often coincides with hybridization and shifts to asexual reproduction involving megagametophytes, known as ‘gametophytic apomixis’ (hereafter apomixis; Campbell and Dickinson 1990, Talent 2009, Dickinson 2018). In this plant family, an increase in the chromosome number set occurred in at least 32 of 85 genera (Kalkman 2004). Hawthorns (*Crataegus* L.) are one such genus. It has attracted attention for centuries due to its economic and ecological significance, exemplifying the phenomena of hybridization, polyploidy, and apomixis, and exhibiting remarkable morphological variation. Depending on the species concept, between 150 and 1200 species have been described (Christensen 1992). Currently, over 200 are widely recognized (Phipps 2015), but systematic and evolutionary relationships of many species and species groups remain poorly understood. Blurred species boundaries and morphologically intermediate individuals complicate their classification (Dickinson et al. 2007).

Documented occurrences of various ploidy levels in *Crataegus* (Muniyamma and Phipps 1979, Ptak 1986, 1989, Smith and Phipps 1988) correlate with their reproductive strategies. Diploid *Crataegus* species primarily reproduce sexually, producing genetically variable offspring (Muniyamma and Phipps 1985, Lo et al. 2009, Vašková and Kolarčik 2019), while polyploid species often employ apomixis leading to clonal offspring. Extensive research in North America (Talent and Dickinson 2005, 2007b, Lo et al. 2009, 2010, Zarrei et al. 2014, Dickinson et al. 2021) has modelled polyploidization processes, hybridization, and parentage of apomictic allopolyploids. In contrast, comprehensive, multidisciplinary investigations of European hawthorns are lacking. Recent studies on European *Crataegus* (Vašková and Kolarčik 2019, Kolarčik et al. 2022) have begun to explore their relationships and reproductive modes using flow cytometry (FCM; Suda et al. 2007) and flow cytometric seed screening (FCSS; Matzk et al. 2000, Matzk 2007, Dobeš et al. 2013). FCSS enables simultaneous determination of embryo and endosperm ploidy levels, serving as the basis for reproductive mode detection.

**Figure 1:**
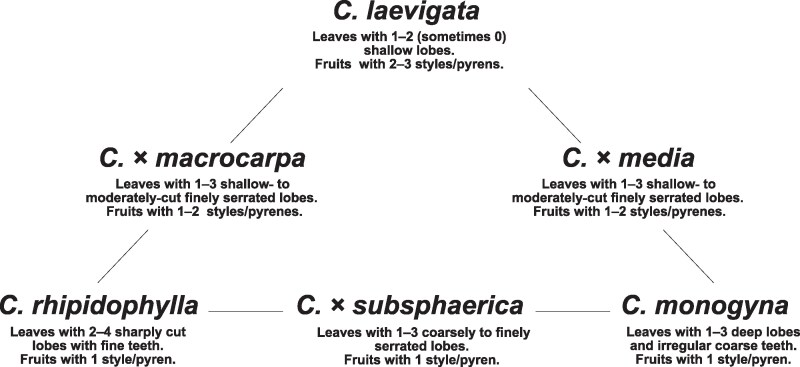
Schematic diagram of relationships among the studied *Crataegus* species and their interspecific hybrids. Beneath each taxon name the diagnostic morphological traits according to Christensen (1992). For detailed comparison see Christensen (1992).

In Central Europe, three native and widespread hawthorn species occur—*C*. *monogyna* Jacq., *C*. *laevigata* (Poir.) DC., and *C*. *rhipidophylla* Gand. The latter can be further divided into at least two varieties. *Crataegus rhipidophylla* var. *rhipidophylla* and *C*. *rhipidophylla* var. *ronnigeri* (K. Malý) Janjic (=*C*. *rhipidophylla* var. *lindmanii* (Hrabětová) K. I. Chr.) (Christensen 1992). The second of these varieties, *C*. *rhipidophylla* var. *lindmanii*, is occasionally treated as a subspecies or even as the separate species *C*. *lindmanii* Hrabětová. The main three species differ in their ecological preference. *Crataegus laevigata* and *C*. *rhipidophylla* are shade-tolerant and can persist in forest habitats, while *C*. *monogyna* prefers open, sunlit sites (Depypere et al. 2006, Oklejewicz et al. 2013). Nevertheless, they may co-occur, producing the putative hybrids *C*. *×*  *subsphaerica* Gand. (*C*. *monogyna* × *C*. *rhipidophylla*), *C*. *×*  *macrocarpa* Hegetschw. (*C*. *rhipidophylla* × *C*. *laevigata*), and *C*. *×*  *media* Bechst. (*C*. *monogyna* × *C*. *laevigata*) that often exhibit intermediate morphology (Fig. 1). Although the parental species are generally well defined (Christensen 1992), determination of hybrids is challenging due to morphological variability and potential introgression (Christensen 1982, Gosler 1990, Depypere et al. 2006). Detailed morphometric analyses (Christensen 1982, Depypere et al. 2006, Kuhn et al. 2020) have highlighted traits critical for delimitation, such as floral style number, leaf shape, basal leaf lobe size, and fruit dimensions and proportions. These species also differ in ploidy levels. *Crataegus laevigata*, *C*. *monogyna*, and *C*. *×*  *media* are typically diploid (2*n* = 34), whereas *C*. *rhipidophylla* occurs as diploid, triploid, and tetraploid (2*n* = 34, 51, 68; Gladkova 1968, Baranec 1986, Ptak 1986). The hybrid *C*. *×*  *macrocarpa* can be either triploid or tetraploid, and *C*. *×*  *subsphaerica* can be diploid or tetraploid (Baranec 1986, Ptak 1986, Lippert 2006).

To date, our understanding of hybridisation among *C*. *monogyna*, *C*. *laevigata*, and *C*. *rhipidophylla* in Central Europe largely relies on morphological evidence, with limited genetic data. Although nuclear microsatellite markers (nuSSRs; nuclear simple sequence repeats) have been employed (Fichtner 2022), morphological identifications have generally taken precedence over genetic findings.

Here, we investigated hybridization, polyploidy, and apomixis in Central European *Crataegus* by examining ten populations in Austria and Slovakia, where all three species and their hybrids co-occur using genotyping, ploidy estimation, assessment of the mating system, and morphological analyses. Species identification was based on genetic groupings (genetic clusters) derived from nuSSRs analyses, providing a robust framework for comparing morphological and reproductive variation. This is thus the first Central European *Crataegus* study that delineates these species based on genetic data. The applied nuSSR set was newly developed from *C*. *monogyna* and *C*. *laevigata* genomic sequences. Ploidy was determined via FCM of leaves, and reproductive modes were identified using FCSS. We adopted the most robust nomenclature (Christensen 1992), aiming to recognize three parental species (*C*. *laevigata, C*. *monogyna, and C*. *rhipidophylla*) and their hybrids (*C*. *×*  *macrocarpa, C*. *×*  *media, and C*. *×*  *subsphaerica)*, without further differentiation (e.g. treating *C*. *lindmanii* as a separate species as in Kerényi-Nagy 2014). Morphological analyses followed classical fruit morphometry, while the leaf shape was assessed using geometric morphometry (Rohlf and Marcus 1993), a method applied in genera like *Quercus* L. and *Acer* L. (Jensen et al. 1993, 2002). This approach, particularly the landmarking, has also been used for American *Crataegus* taxa (Canché-Delgado et al. 2011, Piedra-Malagón et al. 2016).

By integrating genetic, morphological, and cytological approaches, we addressed the following questions: (i) Can genetic markers delineate species and reveal geographic structure? Are hybrids present, and to what extent? (ii) How do observed ploidy levels of mother individuals correlate with their reproductive modes and ploidies of their offspring? What is the reproductive mode of the hybrids? (iii) Does the leaf and fruit morphology of parental species and hybrids correspond to the separation recovered by the genetic analyses? Finally, we discussed the results in relation to the observed genetic patterns, the distribution and abundance of parental species and hybrids, and their reproductive behaviour and morphological diversity. We concluded by addressing the challenges and options for morphological identification in genetically admixed species and hybrids.

## Material and methods

### Sampling

In autumn 2020 (September–October), samples were taken from ten localities in Austria and Slovakia that had been reported to contain all three *Crataegus* species (*C*. *laevigata*, *C*. *monogyna*, *C*. *rhipidophylla*), as stated in Sołtys-Lelek et al. (2012, 2013, 2014) and itineraries of a ‘mapping campaign’ (unpublished data from the current scheme of ‘Mapping the Flora of Austria’, coordinated by H. Niklfeld and L. Schratt-Ehrendorfer, University of Vienna) (Table 1, Supplementary Table S1). Within each locality, 30–33 individuals representing a wide range of morphological diversity in leaves and fruits—including *C*. *laevigata*-like, *C*. *monogyna*-like, *C*. *rhipidophylla*-like individuals, and intermediates between them—were chosen, totalling 316 individuals. Twigs of flowering shoots and short vegetative shoots were collected for morphometric analyses and later preserved as herbarium specimens at the Austrian Research Centre for Forests (BFW) in Vienna, Austria. Additionally, up to 20 leaves were taken per tree. One portion of the fresh leaves was placed directly in silica gel for DNA extraction and genotyping, while the other was stored in a fridge after each field visit for flow cytometric measurements. Where available, fruits were collected in paper bags and left to dry at room temperature for FCSS.

**Table 1:** List of the *Crataegus* localities.

Locality	Code	Country	Longitude	Latitude	Altitude (m)	Area (m^2^)	No. of samples
Michaelerberg	Pop 0	AT	48°14′35.8”N	16°17′03.5”E	318	6 475	33
Kaltenleutgeben	Pop 1	AT	48°06′59.9”N	16°12′53.3”E	446	10 950	33
Kahlenberg	Pop 2	AT	48°16′24.3”N	16°20′32.5”E	329	13 406	32
Rosental	Pop 3	AT	48°01′16.3”N	16°09′02.9”E	420	38 563	30
Sophienalpe	Pop 4	AT	48°14′27.0”N	16°13′23.6”E	476	13 276	30
Pleše	Pop 5	SK	48°48′48.9”N	19°14′45.3”E	865	92 922	31
Vlačuhovo	Pop 6	SK	48°50′05.1”N	19°15′06.3”E	940	33 394	32
Jasenie	Pop 7	SK	48°49′35.5”N	19°28′42.7”E	555	139 122	32
Jakub	Pop 8	SK	48°45′57.8”N	19°08′29.5”E	414	74 268	31
Horná Lehota	Pop 9	SK	48°50′52.0”N	19°32′28.4”E	662	8 150	32

### Plant genotyping

For development of nuSSRs, we used sequences of two *C*. *laevigata* samples (Cr096BGBM, Biosamples: SAMN45047645 and Cr208Wiss, SAMN45047646) and one sample of *C*. *monogyna* (Cr237BIN, SAMN45047647 stored in the SRA with Bioproject number PRJNA1190510) derived from Hyb-Seq data (a mixture of target-enriched next-generation sequencing libraries and unenriched libraries in a 2:1 proportion). To remove sequences lacking microsatellites and to exclude plastid and mitochondrial reads from the SSR search, we used the following procedure. First, we merged the forward and reverse reads in Geneious Prime 2020.0.5 (Biomatters Ltd.). Next, we mapped the merged reads to the Malinae481 probe set (Ufimov et al. 2021), which was used to design the baits for the Hyb-Seq libraries, as well as against the chloroplast (NC_031163.1) and mitochondrial (NC_018554.1) genomes. Finally, we retained only reads that did not map to these references for further analysis. The resulting reads were then *de novo* assembled with SPAdes 3.13.0 (Bankevich et al. 2012), and the resulting contigs were imported to the QDD 3.1.2 pipeline (Meglécz et al. 2010) to search for nuSSRs primers. In the first step, microsatellite-containing sequences were extracted. The length of the flanking region on both sides of the microsatellite was set to 200 base pairs (bp), the minimum sequence length to 100 bp, and the minimum repeat number for di-, tri-, tetra-, penta-, and hexa-base motifs for microsatellite detection to 5. In the second step, unique microsatellite sequences were identified using ‘all against all’ BLAST+ 2.11.0 (Camacho et al. 2009). Primer design for these unique nuSSRs was performed with the Primer3 2.5.0 algorithm (Rozen and Skaletsky 2000). PCR product size was set to 90–400 bp, primer size to 18–27 bp, and primer melting temperature from 52 to 62°C with an optimal temperature of 59°C. In the final step, all sequences with successful primer design were checked by BLAST against the NCBI nucleotide database to screen for contamination and verify the taxonomic classification of the best hit. Out of all resulting nuSSR sequences, only those with variable number of SSR motif repetitions between the three *Crataegus* samples used for the nuSSR search were retained. Additionally, the position of each primer sequence on the *Malus domestica* (Suckow) Borkh. cultivar HFTH1 genome (Zhang et al. 2019) was checked to preferentially choose loci covering all 17 chromosomes. In the end, 42 candidate nuSSR primer pairs were selected based on their chromosomal position, repeat length, and similar annealing temperature.

About 40 mg of leaf tissue from 316 sampled individuals, stored previously in silica gel, was frozen in liquid nitrogen and then ground with a Retsch Mixer Mill 301 (Leeds, UK) for 3 min at 26 Hz. The DNA was extracted from leaves with the Invisorb Spin Plant Mini Kit (Invitek, Berlin, Germany) following the manufacturer's protocol with two modifications: the lysis step was extended to 30 min to increase DNA yield, and 4 µl RNase A (Thermo Fisher Scientific, EN0531) was added. The final DNA was dissolved in 30 μl of Elution Buffer D. DNA concentrations were measured using a Nanodrop 1000 spectrophotometer (Thermo Scientific) and subsequently diluted to 5 ng/μl. In total, DNA from 312 samples was successfully extracted.

In the initial tests, all 42 developed primer pairs were amplified in separate reactions and processed by the M13-tailed SSRs method (Steffens et al. 1993, Schuelke 2000). An M13 primer 5′ TGTAAAACGACGGCCAGT 3′ with WellRED D4 fluorescent dye (Beckman Coulter, Fullerton, CA) bound to the 5′ end was used. Firstly, the two unlabelled primers in each PCR reaction consisted of a forward primer with a 5′ M13 tail (TGTAAAACGACGGCCAGT) and a reverse primer (Table 2, Supplementary Table S2). The Qiagen multiplex PCR kit (Qiagen, Hilden, Germany) was used for the preparation of 10 µl PCR mix consisting of 5 µl of Master mix, 3.4 µl ddH_2_O, 0.2 µl of each SSRs primer, 0.2 µl of M13 primer, and 1 µl of DNA (5 ng/µl). The PCRs were performed in a T100^TM^ Thermal Cycler (Bio-Rad, Hercules, CA, U.S.A.) in a 96-well plate format with an initial denaturation at 95°C for 15 min, followed by first 25 cycles of 94°C for 30 s, primer annealing temperature (54–59°C) for 1 min, and an extension step at 72°C for 1 min, and then another 10 cycles of 94°C for 30 s, 53°C for 1 min, and 72°C for 1 min, with a final elongation step at 60 °C for 30 min. The resulting PCR product (1 µl) was mixed with 31.25 µl of ddH_2_O and 0.52 µl of 400-bp size standard (Beckman Coulter, Brea, CA, USA) and then separated by the CEQ 8000 Genetic Analyser (Beckman Coulter) with the FRAG-3 run method. The obtained alleles were scored with the software GeneMarker 1.8 (SoftGenetics LLC, PA, USA).

**Table 2:** Final list of 22 *Crataegus* nuSSR markers developed and used for genotyping.

Primer name	Forward sequence with M13 tail	Reverse sequence	Motif	Ta F	Ta R	bp	MP	Dye	C (µM)	GenBank*	Chromosome*
**gr1585_7**	TGTAAAACGACGGCCAGTGACGGAGGCAAACACAAACA	GCACACTCCATCTTCGCTTC	(AG)18	59	59	255	1	D2	0,03	CM014058.1	10
**gr1712_4**	TGTAAAACGACGGCCAGTTGCGCTGGAGTTGTACTACA	AAACCGGCTGCAGTAATTCC	(AGC)11	59	59	293	1	D3	0,03	CM014059.1	11
**gr1662_4**	TGTAAAACGACGGCCAGTGCGTTTGCACCATCCTCAAT	GCCGAGTCCATTGTTCGTAC	(AT)14	59	59	265	1	D4	0,03	CM014056.1	8
**gr2371_7**	TGTAAAACGACGGCCAGTGGTTGATGGGTTGAGGGAGA	CATCAGTACAGTACCAAGCCAAA	(GA)18	59	59	363	1	D4	0,03	CM014060.1	12
**gr521_2**	TGTAAAACGACGGCCAGTCTGCAGACCAAGCAATTCGA	TCCACAGAGCAATCCAGACC	(CT)7	59	59	151	1	D4	0,03	CM014051.1	3
**gr2170_3**	TGTAAAACGACGGCCAGTCTCTCCTTCCACGAGTCAC	ACATCCACTCCTTCCTCAGC	(TC)14	59	59	293	2	D2	0,05	CM014049.1	1
**gr2259_5**	TGTAAAACGACGGCCAGTCAAACATCCGTTTTACCCTAC	ATGAGGTGGAGGGATCGTTC	(CT)16	58	59	258	2	D2	0,05	CM014059.1	11
**gr2206_7**	TGTAAAACGACGGCCAGTGGCAACATGTTTACCGTGGT	AGGAGGCCAACTATGAACCA	(AT)16 (GT)21	59	58	284	2	D3	0,08	CM014058.1	10
**gr2253_3**	TGTAAAACGACGGCCAGTTGCAACTGGAATCGCAACAA	ACAGGAATGCGACCTCATCA	(TGC)8	59	59	170	2	D3	0,04	CM014058.1	10
**gr1604_3**	TGTAAAACGACGGCCAGTCCGTGGCTTGTCACTAATTGT	CTCTGTCGTGAATGCCAGTG	(GA)6	59	59	258	2	D4	0,03	CM014058.1	10
**gr2231_3**	TGTAAAACGACGGCCAGTGGGATGGTCTCCTCTCATGG	AGCTGTTGAGATTCACACGT	(GT)9 (GA)16	59	57	295	2	D4	0,05	CM014055.1	7
**gr1736_6**	TGTAAAACGACGGCCAGTTGAGGAGGAGGAGAGAGAGG	AAGACACGAGCCGAAGAGAA	(CT)24(AT)10(TC)6	59	59	175	3	D2	0,11	CM014064.1	16
**gr1630_3**	TGTAAAACGACGGCCAGTACACACACCTTCTCTCTTCTGT	AGCTAGCTCTTGGTGTAGCTA	(CT)17	59	59	134	3	D3	0,10	CM014054.1	6
**gr2305_3**	TGTAAAACGACGGCCAGTTTTCCGCGCCACAAATCAA	TGCGGACGTAGCAGTAGTAG	(AG)7	59	59	275	3	D3	0,05	CM014065.1	17
**gr1597_3**	TGTAAAACGACGGCCAGTCAGTGATCCAGGAGGCCA	AATAAGCTAAGATGGCCTCACC	(GA)15	59	59	279	3	D4	0,05	CM014053.1	5
**gr1774_3**	TGTAAAACGACGGCCAGTTAACCACAACTCGACCGCTA	GTGGCATTTTGGAGAAGGGC	(TC)16	59	59	165	3	D4	0,03	CM014064.1	16
**gr852_3**	TGTAAAACGACGGCCAGTTAAGTTCTGCTGCTCGTCCA	TCGGGCACTTTCAGGAACT	(GA)6	59	59	342	3	D4	0,04	CM014063.1	15
**gr1731_3**	TGTAAAACGACGGCCAGTACCCTATTCTTATTCCTACATTTCTCA	GGTGGAGTCGGTTACTGGAA	(AG)16	58	59	151	4	D2	0,05	CM014052.1	4
**gr989_3**	TGTAAAACGACGGCCAGTAGCTGGATGCAACACTGTTG	AGCTGTCAAGGTAGTAGCCA	(TA)13(CA)8	59	58	139	4	D3	0,03	CM014051.1	3
**gr1572_3**	TGTAAAACGACGGCCAGTAGTTTACGGCGGTGATGAGG	CCTTCCTTCACCTTTCATGACC	(GA)15	59	59	283	4	D4	0,03	CM014051.1	3
**gr1601_3**	TGTAAAACGACGGCCAGTGAGATGTATGGGACGCGGTA	CCCTCTCACCCACTTGGAAT	(GA)13	59	59	179	4	D4	0,01	CM014053.1	5
**gr823_2**	TGTAAAACGACGGCCAGTGCTTTGTGTTCTGCCTTGGA	AGTTCCAAATGGGCAGTTCC	(TC)7	59	58	309	4	D4	0,05	CM014050.1	2

Annealing temperature in °C of forward and reverse primer (Ta F, Ta R), length of PCR product (bp), number of PCR multiplex (MP), WellRED fluorescent dye used for PCRs and fragment analysis (Dye), final concentration of the primer (the same for forward and reverse primer) in PCR multiplex (C (µM)), locus name in *Malus domestica* cultivar HFTH1 (GCA_004115385.1) in GenBank database (GenBank*), chromosome on which the locus occurs in *M*. *domestica* (Chromosome*).

As a result of the testing, 22 microsatellite primer pairs were selected based on peak readability, polymorphism, and reproducibility. These were then combined into four multiplex sets (Table 2, column MP). All forward primers from the selected subset of markers were 5′-fluorescence labelled with WellRED D4, D3, or D2. For the PCR multiplex reactions, the Qiagen Type-it Microsatellite PCR kit (Qiagen, Hilden, Germany) was used with a mixture of the primers specific for each multiplex. The 10 µl of PCR mix was prepared from 5 µl of 2× Type-it Multiplex PCR Master Mix, 2 µl of ddH_2_O, 2 µl of primer mixture, and 1 µl of DNA (5 ng/µl). The concentration of each primer is given in Table 2. The amplification was carried out in a 384-well plate format in a Labcycler Basic (SensoQuest, Göttingen, Germany) with the programme: 95°C for 15 min, followed by 35 cycles of 94°C for 1 min, with the same annealing temperature for all the multiplexes 59°C for 1 min, 72°C for 1 min, and a final extension step at 72°C for 10 min. As 1 µl of the PCR product showed such a strong signal, it was diluted 10 times with ddH_2_O for the final runs of all samples before fragment analysis. The mix was then prepared similarly as before with 1 µl of the diluted PCR product, 31.25 µl of ddH_2_O, and 0.52 µl of 400-bp size standard, and samples were processed with the CEQ 8000 Genetic Analyser. In total, six samples failed and were excluded from the following analysis. For each SSR marker and multiplex, a panel was prepared in the software GeneMarker, and the alleles of all samples were scored semi-automatically, with manual checking after automated scoring. The output of the programme was a matrix of allele sizes. Only unambiguous alleles between 100 and 500 bp were considered for subsequent analyses. In the case of polyploids, each observed allele was recorded only once. The final matrix of allele sizes with unknown allele dosage for polyploid samples consisted of 306 individuals (Supplementary Table S3) and was further processed in the software GenoDive 3.06 (Meirmans 2020). Here, genetically identical individuals (ramets) were detected per population using default settings with the threshold of genetic distance of 22 measured as the smallest number of mutation steps that is needed to transform the genotype of one individual into the genotype of the other under the stepwise mutation model. This threshold was chosen based on a histogram of the frequency distribution of the distances (Supplementary Figure S1). Only unique genotypes (genets) were selected for the subsequent analyses, resulting in a total of 214 genets. Allele dosage was then restored for all polyploid genotypes, also in GenoDive.

The genetic clusters (groups) were assessed with the software STRUCTURE 2.3.3 (Pritchard et al. 2000), testing the optimal number of clusters (*K*) from 1 to 10 with each *K* repeated ten times. The ploidy was set as the highest ploidy in the dataset (4), which added missing data for chromosome sets absent in diploids and triploids. An initial burn-in of 1 000 000 generations and an additional 2 000 000 generations of MCMC chains were run after burn-in. The output files were summarized in STRUCTURE HARVESTER 0.6.94 (Earl and Vonholdt 2012), and the optimal value of *K* was defined as the partition where mean delta *K* was the greatest and the results of replicate runs were close to identity (Nordborg et al. 2005; i.e. with a high similarity coefficient between runs (Evanno et al. 2005). The graphical outputs for selected *K*s were generated by the software CLUMPP 1.1.2 (Jakobsson and Rosenberg 2007) and Distruct 1.1 (Rosenberg 2004). The samples in the resulting barplot were ordered according to their assignment percentages. According to the sudden decline in the percentages between genetic clusters, thresholds were chosen to divide ‘pure’ genetic groups from ‘admixed’ samples. The limits were set to maximum 15% of admixture from the other genetic groups to be considered as ‘pure’ genetic group (similarly to those used for *Bolboschoenus* (Asch.) Palla in Píšová et al. 2017; or *Sparganium* L. in Píšová and Fér 2020), and ‘admixed’ genetic groups (with admixture proportion between 15% and 85%). The nuSSRs genetic groups served as predefined groups in subsequent analyses (Supplementary Table S1, column Genetic group). In addition, a triangular plot was prepared based on the assignment percentages of the samples to the main three genetic groups, corresponding to the species as shown in other studies (for AFLPs Nicolè et al. 2007; SSRs Dar et al. 2020; or ISSRs Khan et al. 2021).

Subsequently, we performed a principal-coordinates analysis (PCoA) in R 3.6.3 (R Core Team 2021) to gain further insight into the grouping of the three species and the admixed (hybrid) samples. We first computed a Bruvo distance matrix with the package *‘polysat’* (Clark and Jasieniuk 2011) and then carried out the PCoA using the ‘*ape’* function *pcoa()*. The resulting PC1–PC2 scatter plot was drawn with ‘*ggplot2’* 3.5.1 (Wickham 2016).

In the final step, based on the results from STRUCTURE, each population was described by the identified species and hybrids, and mapped in geographic space to assess the distribution of ramets and genets using the free and open-source software QGIS version 3.16.3-Hannover (QGIS Development Team 2024).

### Ploidy level estimation of maternal plants and flow cytometric seed screen

One fresh leaf per tree was used for flow cytometric measurements. Altogether, 316 sampled individuals were analysed by FCM to determine their ploidy level. Additionally, 1 260 seeds representing 244 individuals [usually 5 fruits per tree, whenever possible; Supplementary Table S1, flow cytometric seed screen (FCSS)] were processed to estimate their reproductive mode. The seeds were manually extracted from fruit stones (pyrenes), and a portion of the embryo along with the entire endosperm were taken from the seeds. Seed tissue or leaves were chopped together with an appropriate volume of internal standard leaf tissue with a razor blade. *Pisum sativum* L. cv. ‘Ctirad’ (2C = 9.09 pg; Doležel et al. 1998) was the internal standard for the leaves, while both *P*. *sativum* and *Carex acutiformis* Ehrh. (2C = 0.82 pg; Lipnerová et al. 2013) were used for the seed measurements. *Carex acutiformis* was preferred for polyploids, as the fluorescence peaks representing endosperm cells were in some cases close to or overlapped with the *P*. *sativum* peak. Sample preparation and measurement followed the procedure described in Ufimov et al. (2021). A CyFlow ML cytometer (Sysmex Partec, Görlitz, Germany) equipped with a 365 nm UV-LED as the source of UV light for DAPI excitation was used for the measurements. The resulting fluorescence histograms were analysed in the software FloMax FCS 2.0 (Sysmex Partec) to determine the ratio of DAPI-stained nuclei in the G1 phase of the cell cycle (G1 peak) relative to the internal standards. Only high-quality, unambiguous, and well defined peaks were further analysed. The distribution of inferred ratios revealed peaks corresponding to different ploidy levels. To establish the ranges corresponding to various ploidy levels separately for leaves, embryo, and endosperm, and two different standards, we applied a Gaussian mixture model with the *‘scikit-learn’* Python module (Pedregosa et al. 2011), defining these ranges as the mean ± 2 standard deviation for each peak and with the lowest range being assigned to the diploid level. The ploidy levels of maternal individuals, embryos, and endosperms were compared to assess the reproductive mode of each tree. By analysing the ploidy ratio of embryo and endosperm in relation to the ploidy of the maternal tree, we inferred the reproductive pathways as described in Matzk (2007). The maternal ploidy levels and the embryo-to-endosperm ploidy ratios were summarized for each species and hybrid to determine their predominant reproductive modes.

### Morphological measurements and statistical analysis of morphological data

**Figure 2:**
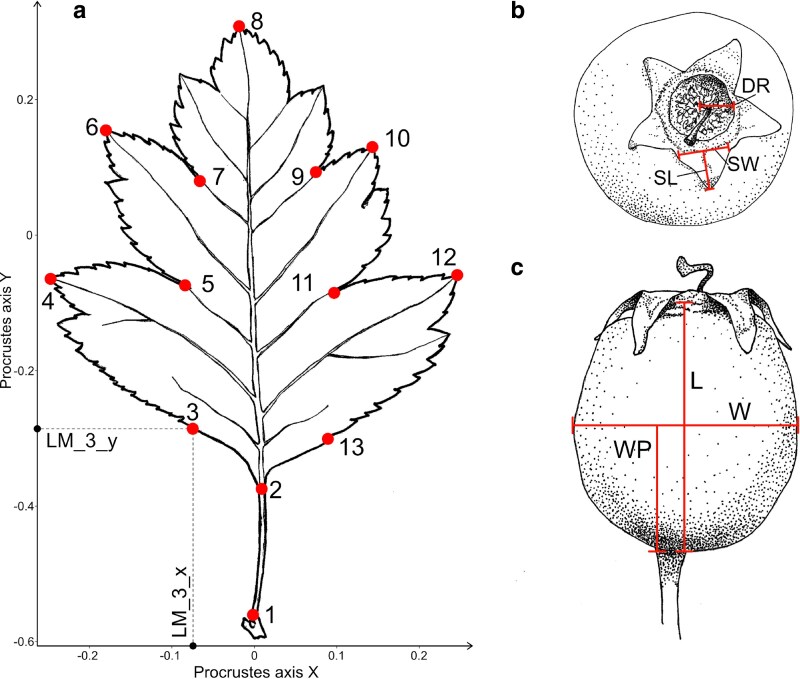
Fruit morphological traits and leaf geometric landmarks analysed in the study. The position of the 13 landmarks on a leaf of *C*. *rhipidophylla*: (a): Juncture where the petiole attaches to the shoot (1), base of the lamina (2), initial serration on the margin of the left basal lobe (3), apex of the left basal lobe (4), left basal sinus (5), apex of the left lobe of the second pair of lobes (6), left-side sinus following the left lobe of the second pair of lobes (7), leaf apex (8), right-side sinus following the right lobe of the second pair of lobes (9), apex of the right lobe of the second pair of lobes (10), right basal sinus (11), apex of the right basal lobe (12), initial serration on the margin of the right basal lobe (13); the position of landmark 3 is projected onto the x and y Procrustes axes as an example. (b) Fruit traits from top view: floral disk radius (DR), sepal length (SL), sepal width (SW). (c) Fruit traits from side view: fruit length (L), fruit width (W), position of the widest part (WP).

Photographs of two to three subterminal leaves from both flowering and short vegetative shoots were taken separately using a standard mobile phone camera. The number of leaves photographed per specimen varied, depending on availability, with a total of 767 leaves from 266 individuals on flowering shoots and 590 leaves from 231 individuals on short shoots (Supplementary Table S1, Fl leaves, Sh leaves). After photographing, landmarks were placed on each leaf in ImageJ 1.53a (Schneider et al. 2012), and their Cartesian coordinates were recorded (Fig. 2a). These coordinates were formatted as TPS files and subsequently processed in MorphoJ 1.07a (Klingenberg 2011), where Procrustes analysis was applied to align and scale the landmark data.

Whenever possible, we randomly selected ten dried fruits from each tree in order to estimate seven fruit traits. In total 1 037 fruits representing 198 individuals were measured (Supplementary Table S1, Fruits). Firstly, the dry fruits were placed in a sealed plastic bag with water, ensuring complete immersion by expelling any trapped air. After soaking for 15–20 h to restore their original size as much as possible, the fruits were observed under a Nikon SMZ18 stereomicroscope equipped with a P2-SHR Plan Apo 1× WD:60 objective lens, C-W10×B/22 eyepiece, and Nikon DS-Fi3 camera. Photographs of each fruit were taken from side and top views and processed with NIS software (Version 5.30.02). Using this software, four traits were measured: the radius of the floral disk (DR), the length (L) and width (W) of the fruit, and the position of the widest part (Fig. 2b and c). In addition, one sepal from each fruit was detached, observed under the microscope, photographed, and its length (SL) and width (SW) were measured with the same software (Fig. 2b). After the measurements, the fruit flesh was removed and the pyrenes were counted (P). The pyrenes were then placed again into a paper bag, and afterwards into a plastic bag filled with silica gel to dry and store them for the flow cytometric analyses.

We first explored the overall *Crataegus* morphospace. Procrustes-aligned landmark coordinates, averaged across all individuals, were subjected to relative-warp analysis with the R package ‘*Morpho’* (Schlager 2017). Prior to analysis, we excluded specimens with uncertain taxonomic assignments and removed within-individual morphological outliers by calculating Procrustes distances from each leaf to its individual mean shape, then excluding leaves exceeding the robust threshold of the median distance + 2 × the median absolute deviation. We then averaged coordinates across all retained leaves per individual and combined the two landmark blocks into a single dataset of 26 landmarks (13 for leaves of flowering shoots and 13 for leaves of short vegetative shoots) and imputed missing data via thin-plate-spline estimation in ‘*geomorph’* (Adams and Otárola-Castillo 2013). Relative warp scores were calculated using the *relWarps()* function with default parameters, as the projection of each specimen’s landmark configuration onto the bending energy eigenvectors. For the fruit dataset, we performed a principal-components analysis with ‘*FactoMineR’* (Lê et al. 2008) on scaled quantitative fruit characters. These exploratory morphospaces were visualized using the first two relative warp axes and the first two principal component axes with ‘*ggplot2*’ (Wickham 2016) to show the spread and overlap of the parental species and the hybrids.

As the exploratory morphospaces illustrate overall variation but do not quantify how distinctly our genetic groups can be separated, we next applied canonical discriminant analysis (CDA) to formally test the discriminatory power of each morphological dataset. CDA was done on all three morphological datasets (fruits, leaves of flowering shoots, and leaves of short shoots) separately followed by a jointanalysis of the combined dataset using the R package ‘*MorphoTools2’* 1.0.1.1 (Šlenker et al. 2022). The X and Y Procrustes coordinates of each landmark were treated as individual traits here. All traits were averaged per tree. The samples assigned by the STRUCTURE analysis as species groups and subgroups were taken as predefined groups for the analyses. Hybrids (genetically admixed samples between STRUCTURE groups) were projected onto the discriminant space of CDA, defined by parental species, as passive samples, so they did not influence the canonical discriminant functions. For the separate analyses of fruits, leaves of flowering shoots, and leaves of vegetative short shoots, only individuals with complete (no missing) data were taken. For the combined analysis, samples with more than 50% missing data were excluded. For the remaining samples, missing data were imputed using the Multiple Imputation by Chained Equations (MICE) technique with the R package ‘*mice’* 3.16 (van Buuren and Groothuis-Oudshoorn 2011). This resulted in a combined dataset representing 251 individuals. Prior to the CDA, all traits were checked for correlations. In all trait pairs with a correlation greater than 0.9, the trait with the lower variance was omitted. The CDA results generated canonical variate scores, which were visualized in scatter plots using ‘*ggplot2’*.

To quantify how well the parental species and the hybrids are discriminated, we subjected the canonical scores to a leave-one-out linear discriminant analysis (LDA) using the function *lda()* from the R package ‘*MASS’* 7.3-60 (Venables and Ripley 2002) with the argument CV = TRUE. In this jack-knife procedure, each individual is classified by an LDA model built from all remaining individuals, yielding unbiased re-classification results. LDA cross-validation was first carried out with the parental species on each individual data block (flowering-shoot leaves, short-shoot leaves, and fruits) as well as the combined dataset. The last run was then performed on the combined dataset that also contained the three hybrids, treating each hybrid as its own group. Overall accuracy and confusion matrices (number of true vs. predicted assignments) were extracted and are reported to illustrate the discriminatory power of each morphological data block.

During CDA and the analysis of classification accuracy, the X and Y Procrustes coordinates of each landmark were treated as individual traits to enable direct assessment of each landmark’s contribution to species discrimination. This coordinate-based approach complements the exploratory relative warp analysis and allows straightforward interpretation of which specific anatomical positions drive taxonomic separation. Because the number of landmarks was small and the number of individuals far exceeded the number of landmarks, it was not necessary to transform the data into warp space to reduce dimensionality (Zelditch 2004). To ensure that this approach yielded results consistent with standard practice, we also performed discriminant analyses using all relative warp scores (obtained from the relative warp analysis in Fig. 5c) as individual traits. Both methods produced nearly identical classification accuracies and separation patterns (results not shown), confirming that coordinate-based discrimination captured the same information while offering more direct anatomical interpretation. Highly correlated traits (Spearman’s *r* > 0.9) were removed to prevent multicollinearity in the discriminant analysis, as such correlations reflect redundant rather than independent information.

## Results

### Genetic groups and population composition

**Figure 3:**
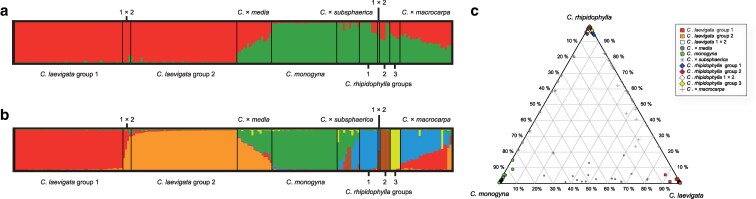
Barplot from the STRUCTURE analysis displaying the assignment of 214 *Crataegus* samples to 11 genetic groups for (a) *K* = 2, and (b) *K* = 6. (c) Triangular plot showing the position of 214 samples based on admixture values from STRUCTURE at *K* = 6.

**Figure 4:**
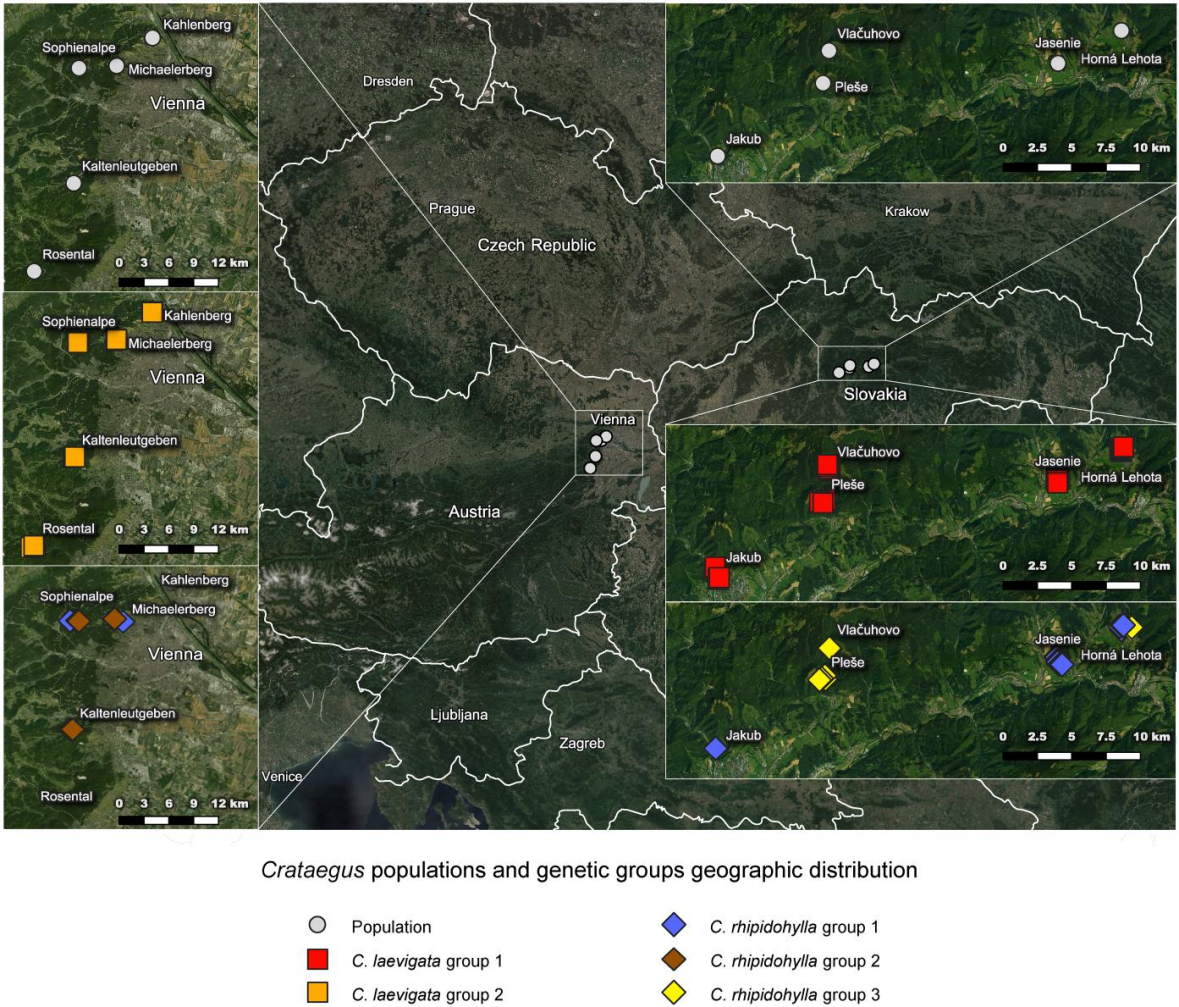
Geographic location of the sampled populations in Austria and Slovakia and their taxonomic identity based on genetic clustering analyses on 22 nuclear microsatellites. *Crataegus laevigata* and *C*. *rhipidophylla* show genetic substructure following a geographic pattern. *Crataegus monogyna*, without any geographic substructure (see Fig. 3), is not shown in this figure.

The genotyped dataset comprising 306 samples (individuals) was processed, and 22 nuSSR loci were scored to create a final matrix of 444 alleles and their sizes. After excluding the ramets belonging to the same clone, 214 samples (genets) were used for the STRUCTURE and PCoA analyses, and the triangular plot. The STRUCTURE analysis identified *K* = 2 as the best solution based on both the highest Δ*K* and the similarity coefficient. This grouping separated only *C*. *laevigata* from the other two species *C*. *monogyna* and *C*. *rhipidophylla* (Fig. 3a). Nevertheless, higher differentiation was achieved with the second highest Δ*K*, *K* = 6 (Fig. 3b), discriminating *C*. *monogyna* from *C*. *rhipidophylla*. Additionally, *C*. *rhipidophylla* was divided into three genetic groups: Group 1, present in both Austria and Slovakia; Group 2 found exclusively in Austrian populations; and Group 3, limited to Slovakian samples (Fig. 4, Supplementary Map S1 and S2). *Crataegus laevigata* was further divided into two genetic groups, whose distribution strictly corresponded to their geographic origin: Group 1 was found in Austria, and Group 2 in Slovakia only. *Crataegus monogyna* formed a distinct group, without division, however well separated from the other species (Fig. 3b). In addition to these six genetic groups representing the species (three for *C*. *rhipidophylla*, two for *C*. *laevigata,* and one for *C*. *monogyna*), a few admixed individuals within both species (*C*. *rhipidophylla,* and *C*. *laevigata*) and a larger proportion of between-species hybrids were identified, including *C*. × *macrocarpa* (57 individuals), *C*. *×*  *subsphaerica* (18 individuals), and *C*. × *media* (17 individuals; Table 3).

**Table 3:** Species and hybrid composition of populations based on 11 genetic groups, and population clonality.

Population	L1	L2	L1 × L2	MED	MON	R1 × R2	R1	R2	R3	SUB	MAC	C. sp.	Pop clonality %
Michaelerberg	—	13/13	1/1	2/2	4/4	—	2/1	11/1	—	—	—	—	**33.3**
Kaltenleutgeben	—	11/9	—	4/4	—	—	—	13/3	—	—	—	5	**42**.**9**
Kahlenberg	—	10/10	2/2	8/8	8/8	—	—	—	—	—	2/2	2	**0**.**0**
Rosental	—	12/12	—	1/1	16/16	—	—	—	—	1/1	—	—	**0**.**0**
Sophienalpe	—	8/8	—	2/2	—	—	1/1	8/1	—	—	11/3	—	**50**.**0**
Pleše	11/11	—	—	—	—	—	—	—	5/2	1/1	13/6	1	**33**.**3**
Vlačuhovo	10/10	—	1/1	—	—	—	—	—	5/2	1/1	15/6	—	**37**.**5**
Jasenie	10/10	—	—	—	1/1	—	16/3	—	—	—	4/2	1	**48**.**4**
Jakub	10/10	—	—	—	3/3	—	1/1	—	—	8/4	9/4	—	**29**.**2**
Horná Lehota	12/12	—	—	—	—	1/1	7/3	—	1/1	7/4	3/2	1	**25**.**8**
R/G per group	**53**/**53**	**54**/**52**	**4/4**	**17**/**17**	**32**/**32**	**1/1**	**27**/**9**	**32**/**5**	**11**/**5**	**18**/**11**	**57**/**25**	**—**	
R/G per species	**111**/**109**					**71**/**20**							
Clonal genets/species	**1**			**0**	**0**	**11**				**5**	**11**	**—**	
Species clonality (%)	**2.7**			**0.0**	**0.0**	**87.3**				**66.7**	**75.4**	**—**	

Number of ramets/number of genets per genetic group or species (R/G per group, R/G per species), number of genets with ramets per species (Clonal genets/species), percentage of ramets within the species (Species clonality (%)), *C. laevigata* group 1 (L1), *C. laevigata* group 2 (L2), *C. laevigata* group 1 × group 2 (L1 × L2), *C.* × *media* (MED), *C. monogyna* (MON), *C. rhipidophylla* group 1 × group 2 (R1 × R2), *C. rhipidophylla* group 1 (R1), *C. rhipidophylla* group 2 (R2), *C. rhipidophylla* group 3 (R3), *C.* × *subsphaerica* (SUB), *C.* × *macrocarpa* (MAC), genetically unidentified samples due to unsuccessful PCR (*C. sp.*), percentage of ramets per population (Pop clonality %). Summary values for ramet and genet numbers and overall clonality per genetic group/species (last four rows) and per population (last column) are shown in bold.

**Figure 5:**
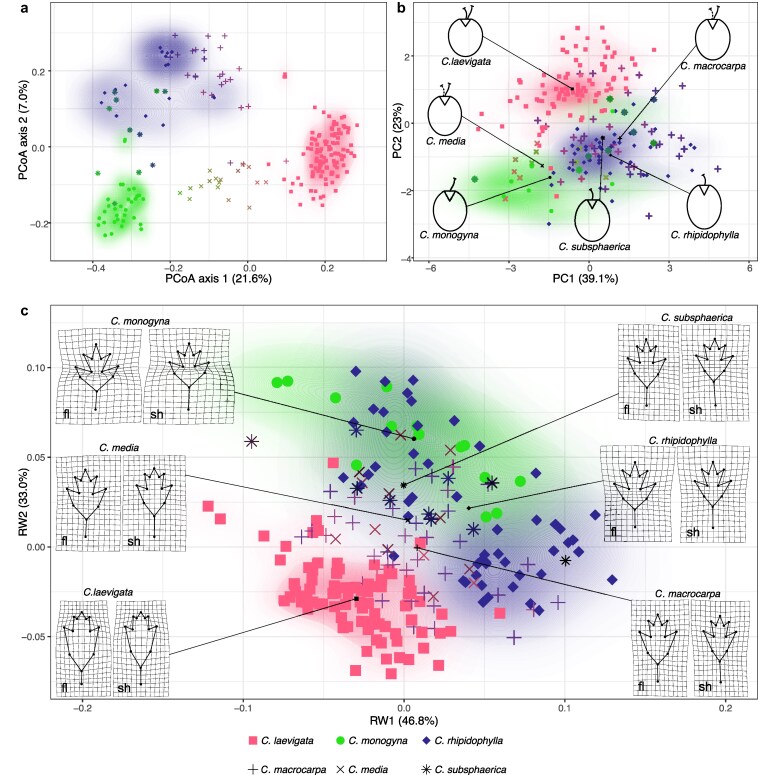
Genetic and morphological position of *Crataegus* species and hybrids. (a) PCoA of microsatellite genotypes, based on Bruvo genetic distances. (b) PCA of fruit morphology. (c) Relative-warp morphospace of leaves. Squares—*C. laevigata* (L), circles = *C. monogyna* (M), diamonds = *C. rhipidophylla* (R); hybrid symbols: plus signs = *C. × macrocarpa* (MAC), crosses = *C. × media* (MED), stars= *C. × subsphaerica* (SUB). Black symbols mark the multivariate centroid of each taxon; adjacent graphics illustrate the corresponding mean fruit outline (b) or thin-plate-spline deformation grids for flowering-shoot (marked as fl) and short-shoot (marked as sh) leaves (c). Background shading in all subpanels shows kernel-density estimates of point distribution for the three parental species, with colour intensity reflecting local concentration in PC/RW space.

In the two-dimensional PCoA plot, the parental species were clearly separated, with no overlap. Notably, two *C*. *monogyna* samples were positioned closer to *C*. *rhipidophyll*a, which displayed the largest spread in the plot (Fig. 5a). The hybrid *C*. × *media* exhibited a distinct intermediate position between its parental species, *C*. *laevigata* and *C*. *monogyna,* a pattern that was less pronounced in the other two hybrids, *C*. × *macrocarpa* and *C*. × *subsphaerica*. In the case of *C*. × *subsphaerica*, most hybrids overlapped with *C*. *rhipidophylla*, while fewer hybrids occupied an intermediate position or even one overlapping with the second parent, *C*. *monogyna*. Similarly, in *C*. × *macrocarpa* hybrids, *C*. *rhipidophylla* had a stronger influence, as the majority of hybrids clustered closer to this species rather than exhibiting a fully intermediate position between *C*. *rhipidophylla* and *C*. *laevigata*. Similar patterns were observed for the hybrids in a triangular plot, based on percentages of sample assignments resulting from STRUCTURE at K = 6 displaying a wide spectrum of admixture between the parental species (Fig. 3c).

When examining individual populations for species and hybrid composition based on the genetic group assignments, the number of identified species and hybrids varied across populations (Table 3, Supplementary Maps S1 and S2). All three paternal species were present in only three locations: Michaelerberg (AT), Jasenie (SK), and Jakub (SK). Michaelerberg had the smallest sampling area of 6 475 m², while Jasenie had the largest with 139 122 m², and Jakub’s sampled area was 74 268 m². The remaining seven populations comprised only two parental species: *C*. *laevigata* emerged as the most common species, being found in all 10 surveyed populations, while *C*. *rhipidophylla* or *C*. *monogyna* were always the second additional species; *C*. *rhipidophylla* was identified in 8 out of 10 populations, and *C*. *monogyna* in 5 out of 10. The high occurrence of *C*. *laevigata* (111 individuals) and *C*. *rhipidophylla* (71 individuals) in the dataset, along with their abundance within the individual populations, coincided with a frequent presence of their hybrid, *C*. × *macrocarpa,* found in 7 out of 10 populations. The hybrids *C*. × *media* and *C*. × *subsphaerica* were both found in 5 populations. *C*. × *media* individuals were found in all the Austrian populations, but they were absent in Slovakia, although in two Slovakian populations (Jasenie and Jakub) both parental species were collected. Interestingly, almost all *C*. × *subsphaerica* individuals (17 out of 18) were found exclusively in Slovakia. However, it is important to note that the PCR amplification failed in 10 collected samples, preventing their classification.

When estimating clonality within the studied samples, two main patterns of clonality emerged in the parental species and their hybrids (Table 3). No ramets were detected among the 32 samples of *C*. *monogyna*, and only three individuals of the same genotype were identified within a total of 109 *C*. *laevigata* genets (Supplementary Table S1, column Genotype). In their hybrid, *C*. × *media*, no ramets were detected. In contrast, 11 out of 20 *C*. *rhipidophylla* genets were clonal, representing 62 out of 71 samples (87.3%). Hybrids with *C*. *rhipidophylla* showed similar patterns: in C. × *macrocarpa*, 11 out of 25 unique genotypes were clonal (43 out of 57 samples, 75.4%), and in *C*. × *subsphaerica*, 5 out of 11 genotypes (12 out of 18 samples, 66.7%) were clonal. The highest extent of clonality among collected samples was observed within the population Sophienalpe (AT) that reached up to 50%. In two populations (Kahlenberg and Rosental, AT), which contained *C*. *laevigata*, *C*. *monogyna*, but not *C*. *rhipidophylla*, clones were missing. The maximum distance between ramets increased with their number within a clone. The following maximum geographic distances of sampled clonal individuals were observed across the different species and hybrids: 5 m in *C*. *laevigata* (3 ramets), 217 m in *C*. *×*  *subsphaerica* (3 ramets), 349 m in *C*. *×*  *macrocarpa* (6 ramets), and 599 m in *C*. *rhipidophylla* (11 ramets).

### Polyploidy and reproductive modes of species

**Figure 6:**
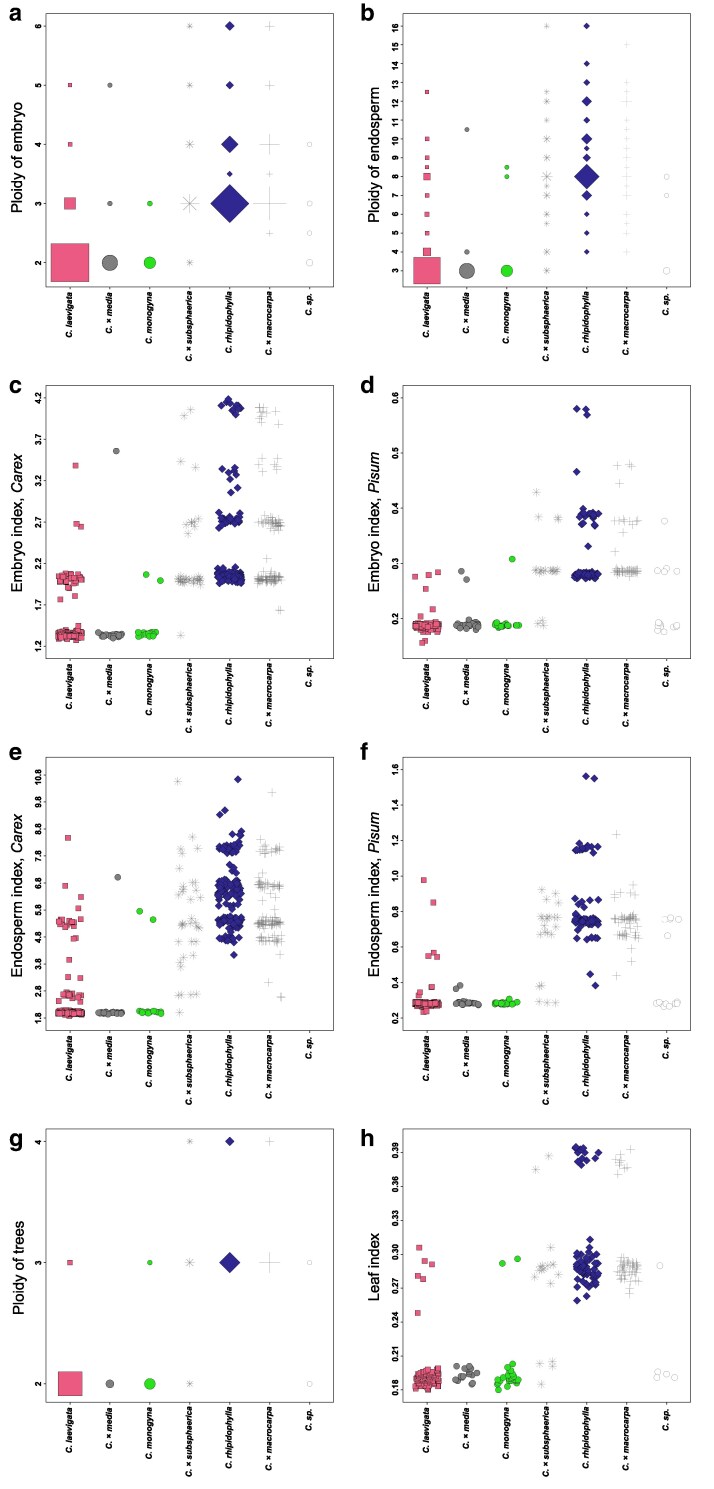
Leaf, embryo and endosperm ploidy and index variation of *Crataegus* species and their hybrids. (a) Ploidy variation of embryos, (b) Ploidy variation of endosperms, (c) Embryo index with *Carex* (FCM ratios of the embryo peaks to the internal standard *Carex acutiformis*), (d) Embryo index with *Pisum* (FCM ratios of the embryo peaks to the internal standard *Pisum sativum* cv. ‘Ctirad’), (e) Endosperm index with *Carex* (FCM ratios of the endosperm peaks to the internal standard *C*. *acutiformis*), (f) Endosperm index with *Pisum* (FCM ratios of the endosperm peaks to the internal standard *P*. *sativum* cv. ‘Ctirad’), (g) Ploidy variation of individuals, (h) Leaf index (FCM ratio of the leaf peaks to the internal standard *P*. *sativum* cv. ‘Ctirad’). Frequencies of ploidy levels in (a), (b), and (g) are represented by different sizes of the symbols.

Our flow cytometric analyses of 306 successfully genotyped individuals confirmed the presence of diploids, triploids, and tetraploids (Table 4, Fig. 6g and h). The measurements revealed that half of these trees were diploid (158 individuals, 52%) accompanied by fewer triploids (124 individuals, 40%) and a small proportion of tetraploids (24 individuals, 8%) (Supplementary Table S1, Leaf ploidy). *Crataegus monogyna* and *C. laevigata* were almost exclusively diploid, in *C. laevigata*, only a few triploids were detected (6 individuals, 5%), and in *C. monogyna*, only 2 triploids (6%) were observed within 31 individuals. Similarly, their homoploid hybrid *C.*  *×*  *media* was exclusively diploid. In contrast, *C. rhipidophylla* was dominated by triploids (56 individuals, 81%) with the remainder being tetraploid (13 individuals, 19%). Polyploidy was also common in the other two hybrid species: all *C.*  *×*  *macrocarpa* trees were polyploid (with 47 triploids and 9 tetraploids among 56 individuals), while *C.*  *×*  *subsphaerica* displayed a mix of ploidy levels (4 diploids, 12 triploids, and 2 tetraploids out of 18 individuals).

**Table 4:** DAPI ratio ranges for each leaf, embryo and endosperm ploidy.

Leaf ploidy (*Pisum*)/number of individuals (percentage of individuals)	2x/158 (52%)	3x/124 (40%)	4x/24 (8%)	Total 306 individuals
Index	0.175–0.206	0.240–0.320	0.370–0.407	

Em ploidy (*Pisum*)	Index	Em ploidy (*Carex*)	Index	Total 1 069 seeds
2x	0.153–0.228	2x	1.053–1.608	536 (50.14%)
—	—	2x–3x	1.609–1.742	3 (0.28%)
3x	0.243–0.318	3x	1.743–2.298	377 (35.27%)
3x–4x	0.319–0.342	—	—	2 (0.19%)
4x	0.343–0.418	4x	2.423–2.978	100 (9.35%)
5x	0.423–0.498	5x	3.053–3.608	25 (2.34%)
6x	0.543–0.618	6x	3.773–4.328	26 (2.43%)

En ploidy (*Pisum*)	Index	En ploidy (*Carex*)	Index	Total 1 069 seeds
3x	0.234–0.322	3x	1.881–2.282	528 (49.39%)
3x–4x	0.323–0.335	—	—	1 (0.09%)
4x	0.336–0.419	4x	2.348–2.912	32 (2.99%)
5x	0.433–0.517	5x	3.058–3.622	5 (0.47%)
5x–6x	0.518–0.519	5x–6x	3.623–3.757	2 (0.19%)
6x	0.520–0.603	6x	3.758–4.322	10 (0.94%)
7x	0.628–0.712	7x	4.428–4.992	67 (6.27%)
7x–8x	0.713–0.716	7x–8x	4.993–5.057	2 (0.19%)
8x	0.717–0.800	8x	5.058–5.622	229 (21.42%)
—	—	8x–9x	5.623–5.872	4 (0.37%)
9x	0.821–0.904	9x	5.873–6.437	44 (4.12%)
9x–10x	0.905–0.920	9x–10x	6.438–6.447	3 (0.28%)
10x	0.921–1.004	10x	6.448–7.012	66 (6.17%)
—	—	10x–11x	7.013–7.159	2 (0.19%)
11x	1.018–1.102	11x	7.158–7.722	6 (0.56%)
12x	1.112–1.195	12x	7.768–8.332	53 (4.96%)
—	—	12x–13x	8.333–8.517	3 (0.28%)
13x	1.213–1.297	13x	8.518–9.082	5 (0.47%)
14x	1.311–1.394	14x	9.128–9.692	2 (0.19%)
15x	1.408–1.492	15x	9.878–10.442	1 (0.09%)
16x	1.512–1.595	16x	10.558–11.122	4 (0.37%)

Ratio (Index) ranges of leaf samples and a standard peak on a DAPI FCM histogram for each ploidy measured with *Pisum*, stating number and percentage of individuals per ploidy (Leaf ploidy (*Pisum*)/number of individuals (percentage of individuals)), ratio (Index) ranges for each ploidy of an embryo and a standard peak of *Pisum* or *Carex* (Em ploidy (*Pisum*), Em ploidy (*Carex*)), ratio (Index) ranges for each ploidy of an endosperm and a standard peak of *Pisum* or *Carex* (En ploidy (*Pisum*), En ploidy (*Carex*)).

During FCSS, we measured both embryo and endosperm ploidy in 1069 seeds collected from 224 individuals. Embryo ploidy ranged from 2x to 6x, while endosperm ploidy varied more widely from 3x to 16x, with several intermediate values observed, particularly in the endosperm (Fig. 6a–f).

The reproductive mode was inferred from the embryo/endosperm ploidy ratio (Table 5). In the largely diploid species *C. laevigata*, nearly all seeds exhibited the expected pattern of sexual reproduction of diploid embryos with triploid endosperm (approximately 89.4% of seeds), while a few seeds displayed triploid (8.1%), tetraploid (0.4%), or even pentaploid embryos (0.2%), with endosperm ploidies ranging from 3x to 12–13x. In *C. monogyna*, all seeds showed the expected diploid embryo/triploid endosperm combination. Likewise, in the homoploid hybrid *C.*  *×*  *media*, nearly all seeds were sexual (2x/3x), with only isolated cases showing a triploid embryo with tetraploid endosperm or a pentaploid embryo with decaploid endosperm.

**Table 5:** Summary of reproductive modes for each ploidy and species with number of seeds with the reproductive mode and the percentage.

	Sexual embryos				Apomictic embryos			Ambiguous
**Maternal individuals**	**2x** L: 428, 91.6% M: 33, 94.3% MED: 49, 94.2% MAC: 2, 1.1% SUB: 4, 6.8%	**3x** L: 18, 3.9% R: 2, 0.9% MED: 2, 3.8% MAC: 2, 1.1% SUB: 6, 10.2%	**4x** L: 1, 0.2% R: 6, 2.6% MAC: 20, 11.4% SUB: 6, 10.2%	**5x–6x** R: 16, 6.8% MAC: 19, 10.8% SUB: 4, 6.8%	**2x** L: 7, 1.5%	**3x** L: 9, 1.9% M: 2, 5.7% R: 155, 66.2% MAC: 106, 60.2% SUB: 33, 55.9%	**4x** R: 31, 13.2% MAC: 15, 8.5% SUB: 3, 5.1%	L: 4, 0.9% R: 24, 10.3% MED: 1, 1.9% MAC: 12, 6.8% SUB: 3, 5.1%
**2x** L: 448, 95.3% M: 31, 88,6% MED: 52, 100% SUB: 10, 16.9%	**♀1+♂1+(**⚇**2+♂1)** L:419, 89.7% M:31, 88.6% MED:49, 94.2% SUB:4, 6.8%	**♀1+♂2+(**⚇**2+♂2)** L:16, 3.4% MED:2, 3.8% SUB:6, 10.2% **♀2+♂1+(**⚇**4+♂1)** L:1, 0.2%	**♀1+♂3+(**⚇**2+♂3)** L:1, 0.2%		**♀2+(**⚇**4+♂2)** L:4, 0.9% **♀2+(**⚇**4)** L:3, 0.6%			**2+(10)** L:1, 0.2% **3+(≥8)** L:2, 0.4% **5+(≥10)** L:1, 0.2% MED:1, 1.9%
**3x** L: 19, 4.1% M: 4, 11.4% R: 188, 80.3% MAC: 150, 85.2% SUB: 41, 69.5%	**♀1+♂1+(**⚇**2+♂1)** L:9, 1.9 M:2, 5.7% **♀∼1.5+♂1+(**⚇**∼3+♂1)** MAC:2, 1.1%	**♀2+♂1+(**⚇**4+♂1)** R:1, 0.45% MAC:2, 1.1% **♀1+♂2+(**⚇**2+♂2)** L:1, 0.2% R:1, 0.45%	**♀3+♂1+(**⚇**6+♂1)** R:5, 2.1% MAC:18, 10.2% SUB:3, 5.1% **♀2+♂2+(**⚇**4+♂2)** MAC:1, 0.6%	**♀3+♂2–3+(**⚇**6+♂2–3)** R:10, 4.3% MAC:11, 6.3% SUB:3, 5.1%		**♀3+(**⚇**6+♂1–4)** L:9, 1,9% M:2, 5.7% R:155, 66.2% MAC:105, 59.7% SUB:32, 54.2% **♀3+(>10)** R:11, 4.7% SUB:2, 3.4% MAC:4, 2.3% **♀3+(**⚇**6)** MAC:1, 0.6% SUB:1, 1.7%		**4+(8)** R:3, 1.3% MAC:5, 2.8% **4–6+(>10)** R:2, 0.9% **5+(7)** MAC:1, 0.6%
**4x** R: 46, 19.7% MAC: 26, 14.8% SUB: 8, 13.6%			**♀2+♂2+(**⚇**4+♂2)** R:1, 0.45% MAC:1, 0.6% SUB:3, 5.1%	**♀4+♂2+(**⚇**8+♂2)** R:6, 2.6% MAC:7, 4% SUB:1, 1.7% **♀3+♂3+(**⚇**6+♂3)** MAC:1, 0.6%			**♀4+(**⚇**8+♂1–4)** R:31, 13,2% MAC:15, 8.5% SUB:3, 5.1% **4+(>12)** R:2, 0.9% MAC:1, 0.6% SUB:1, 1.7%	**6+(≥12)** R:6, 2.6% MAC:1, 0.6%

Rows correspond to the ploidy of the maternal individual, while columns indicate the ploidy of embryos formed in the seeds. Values show the number of seeds and the corresponding percentage. Bold values highlight different ploidy classes (e.g. 2x, 3x, 4x) and indicate embryo–endosperm formation pathways. Reproductive pathways are defined by the ploidy of the egg cell (♀), pollen (♂), and central cell (⚇) contributing to embryo and endosperm formation. Species abbreviations are as follows: *C. laevigata* (L), *C. monogyna* (M), *C.* × *media* (MED), *C. rhipidohylla* (R), *C.* × *macrocarpa* (MAC), *C.* × *subsphaerica* (SUB).

In the polyploid species *C. rhipidophylla*, embryo ploidy varied from 3x to 6x and endosperm ploidy from 4x to 16x, with triploid embryos being the most frequent (∼73% of cases). Some individuals produced only triploid embryos, while others showed mixed embryo ploidies. In the hybrid *C.*  *×*  *macrocarpa*, triploid embryos were slightly less frequent (around 50%), with embryos ranging from 2x to 6x and endosperm from 3x to 15x. Similarly, in *C.*  *×*  *subsphaerica*, embryos ranged from 2x to 6x and endosperm from 3x to 16x. Overall, sexual reproduction was highly predominant in the diploid species (94%–98% of seeds), whereas apomixis was the dominant reproductive mode in *C. rhipidophylla* (with only 10% of seeds resulting from sexual reproduction) and its hybrids, which exhibited a mix of biparental fertilization (∼25%–34%) and apomixis (up to 85% in some cases) (Table 5).

### Morphological variation of fruits and leaves

Seven morphological fruit characters (Fig. 2) were measured on 198 individuals (1 037 fruits in total; Supplementary Table S1). Thirteen morphological leaf landmarks were evaluated on leaves from flowering shoots and vegetative short shoots, on 266 and 231 individuals, respectively (Supplementary Table S1). A combined leaf and fruit dataset was obtained for 251 individuals (see Supplementary Table S9 for the raw morphometric data averaged per individual). When estimating trait correlations, no strong correlations (Spearman *r* > 0.9) were found among fruit traits, so all were included in the CDA. However, for leaf traits, a strong correlation (Spearman r > 0.9) was observed between the *x*-coordinates of the second pair of leaf lobe apices (landmarks 6 and 10) in both shoot types. In separate analyses, the x-coordinate of landmark 10, with the lower variance, was therefore excluded. Similarly, in the combined analysis, the same correlation was noted, but variance was lower for landmark 10 in flowering shoots and landmark 6 in short shoots, leading to their exclusion.

Relative-warp analysis of the imputed 26-landmark composite leaf configuration (13 for flowering shoot leaves and 13 for vegetative short shoot leaves) yielded an ordination in which the first two warps explained 79.8% of the total Procrustes variance (RW1 = 46.8%, RW2 = 33.0%; Fig. 5c). In this space, *C. laevigata* formed a largely discrete cloud, only marginally overlapping the cluster that contained both *C. monogyna* and *C. rhipidophylla*; the points of *C. monogyna* lay completely within the broader spread of *C. rhipidophylla*. The hybrids *C. × media* and *C. × macrocarpa* occupied intermediate positions and overlapped extensively with both parental clusters, whereas *C. × subsphaerica* was almost entirely inside the *C. monogyna–C. rhipidophylla* cluster.

**Figure 7:**
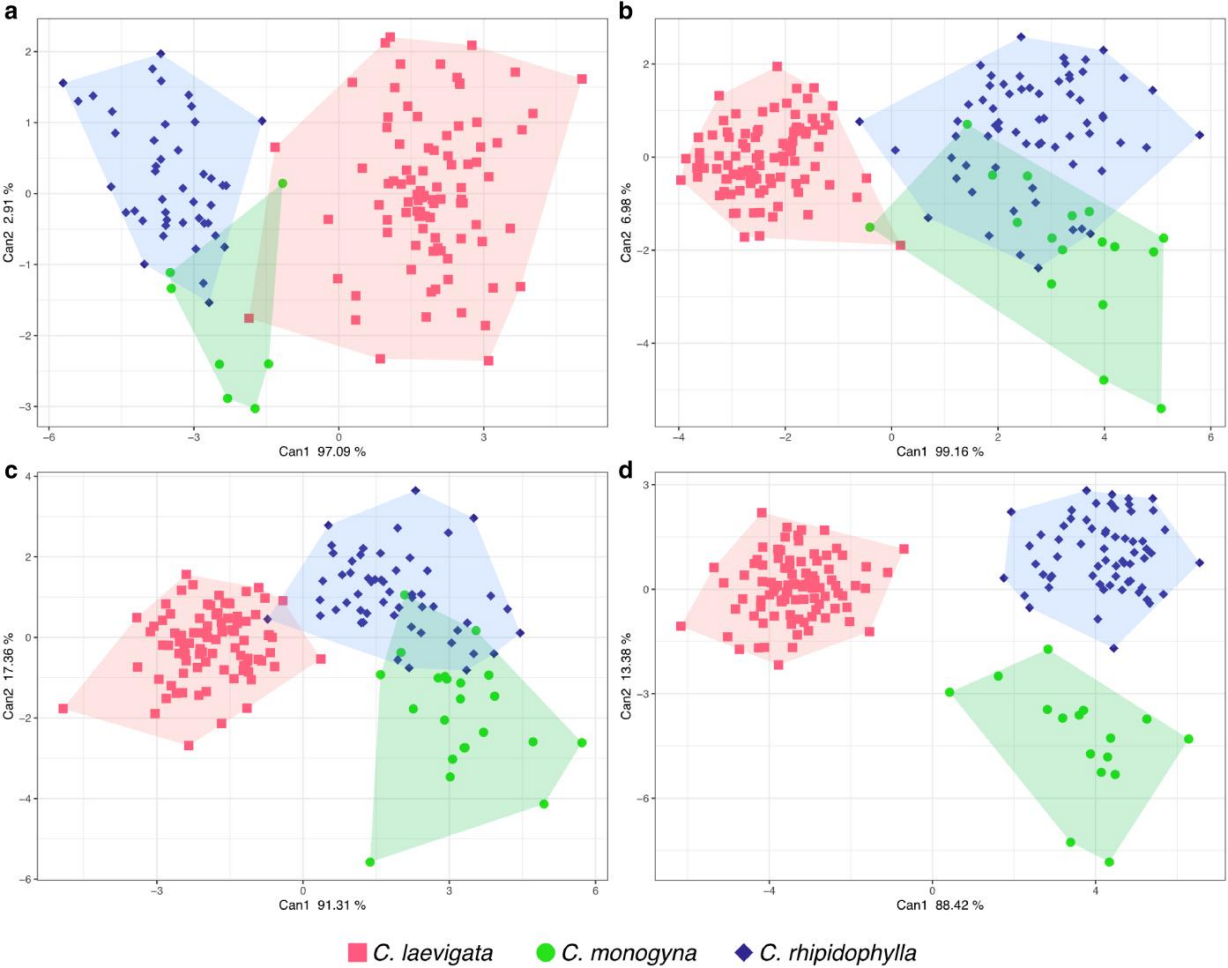
CDA depicting species separation based on 25 leaf and 7 fruit morphological traits. Each plot represents different datasets: (a) Fruits, (b) Leaves from the flowering shoots, (c) Leaves from the vegetative short shoots, (d) and the combined dataset of all traits averaged for each individual. Each point corresponds to an individual, and species are colour-coded: red squares for *C*. *laevigata*, green circles for *C*. *monogyna*, and blue diamonds for *C*. *rhipidophylla*. Only pure species, identified through genotyping, are shown. Shaded convex hulls represent the grouping of individuals for each species, providing a visual separation in the canonical space. Percentage of variance explained by the first and second canonical axes (Can1 and Can2) is indicated on each axis.

For fruits, PCA of the seven size and shape characters produced a comparable pattern: PC1 accounted for 39.1% and PC2 for 21.0% of the total variance (Fig. 5b). Separation along PC2 isolated *C. laevigata* from the other two parental species, while *C. monogyna* and *C. rhipidophylla* formed partially overlapping, opposite extremes along PC1. Hybrids again fell between their respective parents: *C. × media* was largely superimposed on the *C. monogyna* cloud, whereas *C. × macrocarpa* and *C. × subsphaerica* overlapped more with *C. rhipidophylla* than with the second parent. In the fruit CDA (Fig. 7a and Supplementary Table S6), the first two discriminant functions together explained nearly all the observed variation. The first axis, which mainly separated *C. laevigata* from *C. rhipidophylla*, was strongly linked to traits such as the number of pyrenes, fruit length, and sepal length. The second axis—associated with the floral disk radius, fruit length, and the location of the fruit’s widest point—helped to partially distinguish these species from *C. monogyna* (Supplementary Figure S2a).

For the leaf CDA, separate analyses of flowering and short shoots (Fig. 7b and c and Supplementary Table S7) showed that the first two discriminant functions explained almost all the variation. In both shoot types, the same key traits defined the two axes. The first axis was mainly characterized by features such as the positions of the basal lobe apex, the left basal sinus, and the lamina base, which clearly separated *C. laevigata* from the other two species. The second axis highlighted differences in traits like the initial serration on the basal lobe margins and the apex position of the second pair of lobes, further differentiating *C. rhipidophylla* from *C. monogyna* (Supplementary Figure S2b and c).

In the combined analysis (Fig. 7d and Supplementary Table S8), the overall variation was well captured by the first two discriminant functions, and all three species were clearly separated. However, *C. rhipidophylla* and *C. monogyna* remained more similar to each other than either was to *C. laevigata*. Traits most effective at distinguishing *C. laevigata* (associated with the first axis) included the number of pyrenes, the vertical position of the left basal sinus, and the horizontal position of the right basal lobe apex in flowering shoot leaves. Meanwhile, the second axis, which further separated *C. rhipidophylla* from *C. monogyna*, was primarily influenced by the initial serration on the basal lobe margins in short shoot leaves and the apex position of the second pair of lobes in flowering shoot leaves. No additional subdivisions linked to geographic distribution were observed, so the analyses focus on the main species groups.

**Figure 8:**
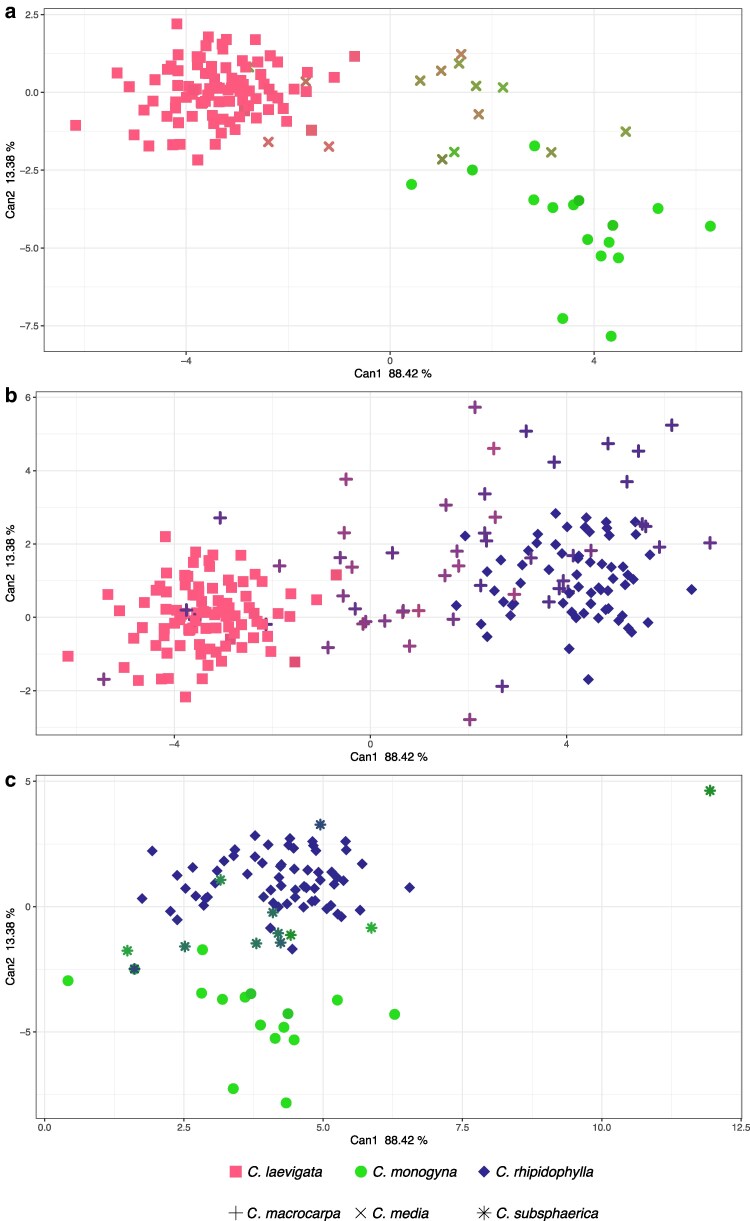
CDA of hybrids and their parental species based on all the combined dataset of all traits (fruits, leaves from the flowering shoots, leaves from the short shoots). Each (a–c) represents a different hybrid, with hybrid individuals plotted alongside their parental species: (a) *C*. × *media*, (b) *C*. × *macrocarpa*, (c) *C*. × *subsphaerica*. The colour gradient of the symbols representing the hybrids corresponds to the percentage of genetic admixture as a result of the STRUCTURE analysis with *K* = 6, blending between the colours of their parental species: red (*C*. *laevigata*), green (*C*. *monogyna*), and blue (*C*. *rhipidophylla*). The axes represent the first two canonical variates (Can1 and Can2), with the percentages indicating the proportion of discrimination explained by each canonical axis.

The CDA revealed some patterns of morphological similarities between hybrids and their parental species based on genetic admixture (Fig. 8). Hybrids showed varying degrees of correlation between their genetic admixture and morphological traits. For *C*. × *media* (Fig. 8a), hybrids with a higher genetic proportion from one parent tended to look morphologically like that parent, displayed by their closer position to that particular parental species in the CDA plot. Thus, hybrids with more genetic admixture from *C*. *laevigata* were closer to the morphospace occupied by *C*. *laevigata*, while those hybrids with more genetic influence from *C*. *monogyna* were positioned nearer to *C*. *monogyna*. However, this pattern was not observed for *C*. × *macrocarpa* (Fig. 8b). Despite varying genetic admixture, the hybrids did not exhibit a clear morphological trend towards either parental species and were scattered across the plot without a consistent pattern. The results of *C*. × *subsphaerica* (Fig. 8c) were also inconclusive. Some hybrids showed a link between genetic admixture and morphological similarity, but the pattern was inconsistent across.

Leave-one-out LDA of the CDA scores (Supplementary Table S10) demonstrated that leaf and fruit variables are highly diagnostic for the three parental species. Classification accuracies were 93% for flowering-shoot leaves, 95% for short-shoot leaves, 96% for fruits, and 99% for the combined character set (Supplementary Table S10A–D). Misassignments were almost exclusively mutual confusions between *C. monogyna* and *C. rhipidophylla*. When the three hybrids were included as additional groups in the combined dataset, the overall accuracy dropped to 75% (Supplementary Table S10E), chiefly because *C. × macrocarpa*, *C. × media*, and *C. × subsphaerica* were variably reassigned to one or both parents, whereas the parental taxa themselves remained correctly classified most of the time.

## Discussion

Here we employed a multifaceted approach, combining genotyping, ploidy level assessment, morphological analyses of fruits and leaves, and mating system evaluation, to investigate how hybridization, polyploidy, and apomixis shape the diversity of Central European hawthorns. The majority of previous genetic studies have focused on other *Crataegus* species, their geographic variation (Lo et al. 2009, Rahmani et al. 2015, Emami et al. 2018, Du et al. 2019), or infraspecific subdivisions (Khiari et al. 2015). Focusing on three European *Crataegus* species (*C*. *laevigata*, *C*. *monogyna*, and *C*. *rhipidophylla*) and their hybrids (*C*. *×*  *macrocarpa*, *C*. *×*  *media*, and *C*. *×*  *subsphaerica*), we sampled individuals from ten populations in Austria and Slovakia where these parental species and their hybrids were known to co-occur. In the field, we collected fruits and branches with leaves from individuals representing a broad range of morphologies, including potential hybrids. Species identification was based on genetic groupings revealed by newly developed nuSSR markers, making this, to our knowledge, the first *Crataegus* study in Central Europe to rely on genetic data as the primary criterion for taxonomic classification. Notably, genetic results sometimes differed from morphological groupings, particularly in hybrids, highlighting the limitations of relying solely on morphology in species determination. Additionally, FCSS enabled us to assess reproductive modes and to detect shifts linked to increasing ploidy levels and hybridization. Overall, our findings underscore the complexity of species boundaries in the genus *Crataegus* and emphasize the need to integrate genetic, morphological, and cytological data to achieve a better understanding of evolutionary relationships and hybridization dynamics. The datasets generated here also provide a foundation for future integrative taxonomic efforts, potentially enhanced by artificial intelligence tools (Karbstein et al. 2024).

### Genetic makeup identifies species, hybrids and geographic distribution

Unlike most previous genotyping studies on *Crataegus* that employed nuSSR markers derived from apple or pear genomes (Zhang et al. 2008, Emami et al. 2018, Güney et al. 2018, Fichtner 2022, Sagbas et al. 2023), we developed a new set of 22 microsatellite markers from *C*. *monogyna* and *C*. *laevigata* genome skimming data to better suit the taxa investigated here.

Our analyses identified six main groups corresponding to the three species (*C*. *laevigata*, *C*. *monogyna*, and *C*. *rhipidophylla*), with further intraspecific subdivisions reflecting their geographic distribution. *Crataegus laevigata* formed two clades, one with samples from Austria and the second with samples from Slovakia. For *C*. *rhipidophylla*, three genetic clusters were found. One was present only in Austria and one only in Slovakia, whereas the third cluster largely consisted of Slovakian samples, though a few samples were found in Austria. Only *C*. *monogyna* formed a uniform genetic group across both Austria and Slovakia. Earlier studies using chloroplast PCR-RFLP and universal angiosperm cpSSR markers found low genotypic variation in *C*. *laevigata* and *C*. *monogyna* across large European sampling ranges (Fineschi et al. 2005). Uniform sequences of *C*. *monogyna* in the *trn*L-*trn*F chloroplast region have been reported in samples from Serbia, Germany, and Greece (Albarouki and Peterson 2007). This lack of variation is not surprising given that plastomes in Rosaceae are rather conserved in gene order and content, and generally differ from nuclear genomes in heritability and mutation rate (Zhang et al. 2017). However, little differentiation within *C*. *monogyna* and *C*. *laevigata* was also detected using nuclear markers such as nuSSR markers for some populations in Germany (Fichtner 2022). Only Ferrazzini et al. (2008) reported genetic divergence among several Italian populations of *C*. *monogyna* using RAPD markers. Without knowledge of the locations of Pleistocene refugia or post-glacial colonization routes for these three species, it remains difficult to interpret the observed patterns. Nonetheless, our newly developed markers provided greater resolution for *C*. *laevigata* and *C*. *rhipidophylla* populations over a relatively small geographic area (distances between localities in different countries ranged from 215 to 265 km), in contrast to the more limited differentiation found in broader-scale studies based on Europe-wide sampling (Fineschi et al. 2005, Albarouki and Peterson 2007, Fichtner 2022).

*Crataegus rhipidophylla* was treated here in a broad sense (*sensu lato*), covering at least two infraspecific taxa (Christensen 1992), and our genetic groups may reflect these subdivisions. For instance, some of our *C*. *rhipidophylla* populations could correspond to *C*. *rhipidophylla* var. *ronnigeri* (syn. *C*. *lindmanii*). In the studied localities, hybrids of *C*. *rhipidophylla* var. *ronnigeri*—*C*. *×*  *macrocarpa* nothovar. *calycina*, *C*. *×*  *subsphaericea* nothovar. *domicensis* (Hrabětová) K. I. Chr.—were also reported (Sołtys-Lelek et al. 2012, 2013, 2014). However, the diagnostic feature distinguishing these varieties/nothovarieties is the presence of erect sepals in mature fruits. Since none of our samples displayed erect sepals, we concluded that *C*. *rhipidophylla* var. *ronnigeri* was absent from our study. Consequently, the relationships between these varieties and nothovarieties remain unclear. Discovered groups within *C*. *laevigata* cannot be attributed to infraspecific taxa, as none are recognized in this species (Christensen 1992).

We observed a wide range of genetic admixture among species and between the Austrian and Slovakian groups of *C. laevigata*. Gene flow among the three *C. rhipidophylla* groups appears virtually absent, indicating that these populations are reproductively isolated from each other. However, gene flow between *C. rhipidophylla* and the other two species (*C. laevigata* and *C. monogyna*) remains ongoing. Individuals displaying admixture from two different species correspond to known hybrids: *C*. *×*  *macrocarpa*, *C*. *×*  *media*, and *C*. *×*  *subsphaerica*. In this study, we defined hybrids as individuals showing admixture levels between 15% and 85%. Such a broad range includes not only first-generation (F1) hybrids, but also subsequent, advanced hybrids and backcrosses. Admixture values of identified hybrids demonstrate gradual distribution across the range, suggesting that F1 hybrids continue to breed with parental species, thereby facilitating ongoing introgression.

Previous morphometric studies in the UK (Byatt 1975, Gosler 1990) documented frequent introgressive hybridization where *C*. *laevigata* and *C*. *monogyna* co-occur, forming hybrid swarms. Byatt (1975) noted that while *C*. *monogyna* maintained its distinct identity, finding a pure *C*. *laevigata* population unaffected by introgression was difficult in southeastern England. Gosler (1990) suggested that differing ecological preferences and habitat disturbance fostered successful hybrid establishment without completely replacing the parental species. Although we also found evidence of introgression, pure species were more common than anticipated. Species boundaries may persist due to ecological differentiation, staggered flowering times—*C*. *laevigata* blooms first, followed by *C*. *rhipidophylla* and then *C*. *monogyna* (Kuhn and Ruprecht 2023)—or genetic incompatibilities and karyotypic differences.

Our sampling strategy focused on capturing a broad range of morphological diversity rather than accurately estimating the true abundance of species and their hybrids. Nevertheless, some patterns emerged: *C*. *laevigata* was found at all sampled sites, followed by *C*. *rhipidophylla* in eight populations and then *C*. *monogyna* predominantly encountered in Austrian populations, with only several individuals found in Slovakia. The hybrid *C*. *×*  *media* appeared only in Austria, whereas *C*. *×*  *macrocarpa* and *C*. *×*  *subsphaerica* were mostly detected in Slovakia. Notably, *C*. *×*  *subsphaerica* was found more frequently near *C*. *rhipidophylla*, while *C*. *×*  *macrocarpa* commonly co-occurred with *C*. *laevigata* rather than *C*. *rhipidophylla*. However, some parental species not collected in our study may still occur at the investigated sites or within a few kilometres, remaining close enough to permit gene flow, as previous authors who reported all taxa at these localities conducted more extensive sampling (Sołtys-Lelek et al. 2012, 2013, 2014).

### Ploidy dictates the mode of reproduction

The ploidy levels detected in our genetically identified species generally matched those reported in the literature. Both *C*. *monogyna* and *C*. *laevigata* were almost exclusively diploid, consistent with previous studies (Gladkova 1968, Baranec 1983, 1986, Ptak 1986). Only a few triploid individuals were encountered in these species, aligning with occasional triploids noted by Gladkova (1968) and Talent and Dickinson (2005). Likewise, *C*. *×*  *media*, commonly regarded as a homoploid hybrid between *C*. *laevigata* and *C*. *monogyna* (Talent and Dickinson 2005), was exclusively diploid here. In contrast, no diploids were identified among our *C*. *rhipidophylla* samples; all were either triploid or tetraploid. Some previously reported diploid counts for *C*. *rhipidophylla* may have been based solely on morphology and could have included misidentified individuals. For instance, the diploid hybrid *C*. *×*  *subsphaerica* (a cross between *C*. *monogyna* and *C*. *rhipidophylla*) could resemble *C*. *rhipidophylla* and may have led to confusion. Still, we do not rule out the presence of diploid forms in other parts of *C*. *rhipidophylla*’s range. A similar geographic pattern occurs in some North American species, such as *C*. *crus-galli*, which is exclusively tetraploid in the north of its range but includes diploid populations at its southern boundary (Talent and Dickinson 2005). By comparison, the other *C*. *rhipidophylla* hybrid, *C*. *×*  *macrocarpa*, was never found as a diploid in our study here.

Our findings shed light on the complex reproductive behaviour observed in *Crataegus*. The homoploid hybrid *C*. *×*  *media*, arising from two diploid parental species (*C*. *monogyna* and *C*. *laevigata*), predominantly reproduces sexually. Nevertheless, it can occasionally be fertilized by either diploid or polyploid pollen, potentially giving rise to polyploid progeny. This scenario also applies to both of its diploid parental species and to occasional diploid individuals of *C*. *×*  *subsphaerica*, which, although hybridogenous, can reproduce sexually as diploids and receive either reduced or unreduced gametes. A striking observation is the occurrence of apomictic reproduction in diploid *C*. *laevigata*—both pseudogamous and autonomous apomixis—although this phenomenon appeared exceedingly rare (only 5 seeds out of 467). Such instances of diploid apomixis have been noted mainly in genera like *Boechera* and *Paspalum* (Hörandl et al. 2024), underscoring how unusual it is in *Crataegus*. Additionally, a few triploid individuals of both *C*. *laevigata* and *C*. *monogyna* exhibited a mixture of sexual and apomictic reproduction.

Results of our study indicate that hybridization does not appear to be a prerequisite of apomixis. For example, *C*. *×*  *media*, though a hybrid, is diploid and does not exhibit apomixis, while polyploid individuals are mostly apomictic. Even triploid individuals of the otherwise sexual parental species (*C. laevigata* and *C. monogyna*) are more likely to switch to apomixis. Consequently, polyploid *C*. *rhipidophylla* and its hybrids are predominantly apomictic, forming embryo sacs without chromosome reduction. While apospory (i.e. embryo sac develops from a somatic cell in the ovule) is considered more common than diplospory (i.e. embryo sac develops from the megaspore mother cell without meiosis) in *Crataegus* (Talent and Dickinson 2007a), our data do not allow us to determine which mechanism is at play. Nevertheless, we detected embryo/endosperm ploidy ratios that suggest rare meiosis-like events in some polyploid individuals, potentially leading to imbalanced gametes and explaining intermediate ploidy levels—a finding consistent with Kolarčik et al. (2022). Furthermore, some polyploid eggs appear capable of accepting pollen with various ploidy levels, resulting in embryos with ploidy exceeding that of the maternal plant. Pollination of unreduced eggs by pollen of any ploidy can yield embryos with ploidy levels higher than four. In fact, we observed pentaploid and hexaploid embryos, but we did not find any adult plants with such high ploidies, suggesting that these embryos either fail to germinate or do not survive in natural populations.

Endosperm formation in studied *Crataegus* typically requires fertilization with one or more sperm cells, rendering apomixis generally pseudogamous, as was found in other studies (Talent and Dickinson 2007a). Fully autonomous endosperm development independent of pollen remains a rare event within the studied samples. Intermediate ploidies are more frequent in the measured seeds at higher endosperm ploidy levels, suggesting that imbalanced sperm cells readily fuse with the central cell of apomictic polyploids to initiate endosperm development. This flexibility arises because paternal contributions in *Crataegus* do not strictly adhere to the typical 2:1 ratio (Talent and Dickinson 2007a). Elevated endosperm ploidy also provides evidence for endoreduplication or polyspermy, where multiple pollen nuclei contribute, potentially creating tri-parental endosperm, as indicated by earlier studies (Vašková and Kolarčik 2019, Kolarčik et al. 2022).

The observed reproductive behaviour helps explain the patterns of admixture. As apomicts, *C*. *rhipidophylla* individuals produce seeds asexually, resulting in offspring that are genetic clones of the mother plant. Through these apomictic seeds, both *C*. *rhipidophylla* and its hybrids actively establish themselves in natural populations. hybridization occurs only under exceptional circumstances—when a rare, successful meiotic event is followed by pollination from a diploid species or when *C*. *rhipidophylla* serves as a pollen source for other diploid species. The near absence of admixture between *C*. *rhipidophylla* populations can thus be attributed to the low frequency of successful embryo formation when accepting pollen from other polyploid individuals, as well as to the reduced viability of seeds carrying embryos of higher ploidies. *Crataegus*  *×*  *macrocarpa* appears to behave similarly to *C*. *rhipidophylla*, but assessing its degree of isolation is more challenging. Variable admixture levels in *C*. *×*  *macrocarpa* may stem from different allele dosage inherited from its polyploid parentage.

In contrast, *C*. *×*  *media*, which reproduces sexually, likely behaves as a typical diploid species. Its ability to backcross with either parental species or even with *C*. *rhipidophylla*—as indicated by individuals displaying additional admixture—maintains gene flow and genotypic diversity. Finally, *C*. *×*  *subsphaerica* shows intermediate reproductive behaviour: some individuals appear capable of sexual reproduction, while others rely mainly on apomixis.

### Morphological variation discriminates species but struggles with hybrid complexity

Morphological analyses based on genetically defined groups—corresponding to the three parental species and their hybrids—combined classical morphometry for fruit and style number with geometric morphometry (Jensen et al. 1993, Rohlf and Marcus 1993) for leaf shape. The use of geometric morphometry, which utilizes landmarks to assess intra- and interspecific variation, has already proven effective for differentiating *Crataegus* species (Dickinson and Phipps 1984, 1986, Canché-Delgado et al. 2011, Piedra-Malagón et al. 2016). Because this method quantifies shape independently of scale, it effectively isolates genuine shape differences from those influenced by environmental factors or ploidy levels. This approach has also outperformed linear morphometric measurements in other plant groups with high morphological variation, such as *Quercus* (Viscosi et al. 2009).

In our analyses, combining seven morphological fruit traits and 13 leaf shape landmarks for three parental non-hybrid individuals yielded clear separation among all three species. These species are also generally well recognized in pure populations where hybrids are absent (Franco 1968, Byatt 1975, Depypere et al. 2006, Sołtys-Lelek et al. 2013, Kuhn et al. 2020). While fruit characteristics and leaves from flowering and short vegetative shoots readily distinguished *C*. *laevigata* from *C*. *rhipidophylla* and *C*. *monogyna*, all traits needed to be considered to fully separate *C*. *rhipidophylla* and *C*. *monogyna*. The latter two share similar one-styled fruits and differ mainly in subtle leaf features, such as serration and the shape of the second pair of lobes. Although most species identification keys emphasize flowering shoot leaves as more stable diagnostic traits (Christensen 1992, Kuhn et al. 2020), we found that leaves from short shoots are at least as informative, particularly for distinguishing *C*. *monogyna* from *C*. *rhipidophylla*. Despite the clear genetic subdivisions detected in *C*. *laevigata* and *C*. *rhipidophylla*, we did not find corresponding morphological groups to match these genetic clusters.

Hybrids pose a greater challenge for identification due to their morphological traits resembling and thus overlapping with one or both parental species. However, the nature of this overlap varies among different hybrid types. In the case of *C*. *×*  *media*, individuals generally displayed intermediate traits, and their resemblance to either parent correlated with their level of genetic admixture. This suggests that successive backcrosses with a particular parent increase morphological similarity to that parent, resulting in a gradual pattern of introgression between *C*. *monogyna* and *C*. *laevigata*. In contrast, *C*. *×*  *macrocarpa* does not exhibit such a clear pattern. Most *C*. *×*  *macrocarpa* individuals resembled *C*. *rhipidophylla* regardless of their genetic admixture level, making them more easily misidentified as *C*. *rhipidophylla*. The relationship between morphology and admixture levels in *C*. × *subsphaerica* is unclear. We did not see a straightforward connection between the degree of genetic admixture and morphological patterns, but some individuals closely resembled one of the parental species. As noted by Sołtys-Lelek et al. (2013), a complex analysis of multiple morphological features, including detailed leaf morphometry, is essential for identifying this hybrid, which shows significant variability.

Relying solely on morphological intermediacy to identify hybrids is problematic, as this intermediate form may represent only a subset of all hybrids. In general, all three hybrids (*C.* × *media*, *C.* × *macrocarpa*, *C.* × *subsphaerica*)—especially those morphologically similar to their parents—are difficult to distinguish accurately and can be easily misidentified as a parental species (Browicz 1972, Byatt 1975, 1976, Christensen 1982, Kuhn et al. 2020). Integrating genetic, morphological, and cytological evidence, as demonstrated in this study, is crucial for accurately classifying *Crataegus* taxa. An integrative taxonomic approach—where molecular data provide a reliable baseline for species delimitation, and traditional morphological and cytological traits refine and confirm these boundaries—can significantly improve the identification process. By combining multiple lines of evidence, researchers can resolve complex taxonomic issues, better recognize hybrid forms, and develop more robust and predictive classification systems.

## Conclusion

Our comprehensive investigation of Central European *Crataegus* highlights the intricate interplay of hybridization, polyploidy, and apomixis in shaping species boundaries and diversity. By integrating genetic, morphological, and cytological data, we distinguished parental species and hybrids, uncovered population-level genetic structure, and delineated patterns of hybridization and introgression. Although morphological traits alone could reliably separate pure species, hybrids often defied simple classification, underscoring the need for a multifaceted approach. The prevalence of apomixis, especially in polyploids and their hybrids, further complicates evolutionary trajectories, influencing gene flow, clonality, and the stability of species boundaries. Our findings lay the groundwork for future integrative taxonomic efforts that combine genomics, morphometrics, cytology, and advanced analytical methods. Such comprehensive strategies will be essential for resolving complex evolutionary histories and refining the taxonomy of *Crataegus* and other challenging plant groups.

## Supplementary Material

plaf067_Supplementary_Data

## Data Availability

The raw genotype, morphometric, and flow cytometry datasets generated during this study are provided as Supplementary Information. All analysis scripts, including R code and input files, are available in a public GitHub repository: https://github.com/rufimov/crataegus-hybrid-zone-analysis/.
