# Supplementary material for: Wide diversity in narrow geographic space: genetic, morphological and ploidy variation in three Central European Crataegus species with emphasis on their reproductive modes
Source: AoB Plants. 2025 Nov 29;18(1):plaf067. doi: 10.1093/aobpla/plaf067 (PMC12818092; doi:10.1093/aobpla/plaf067)
Supplement: plaf067_Supplementary_Data [file plaf067_supplementary_data.zip › Supplementary_figs_maps.pdf]

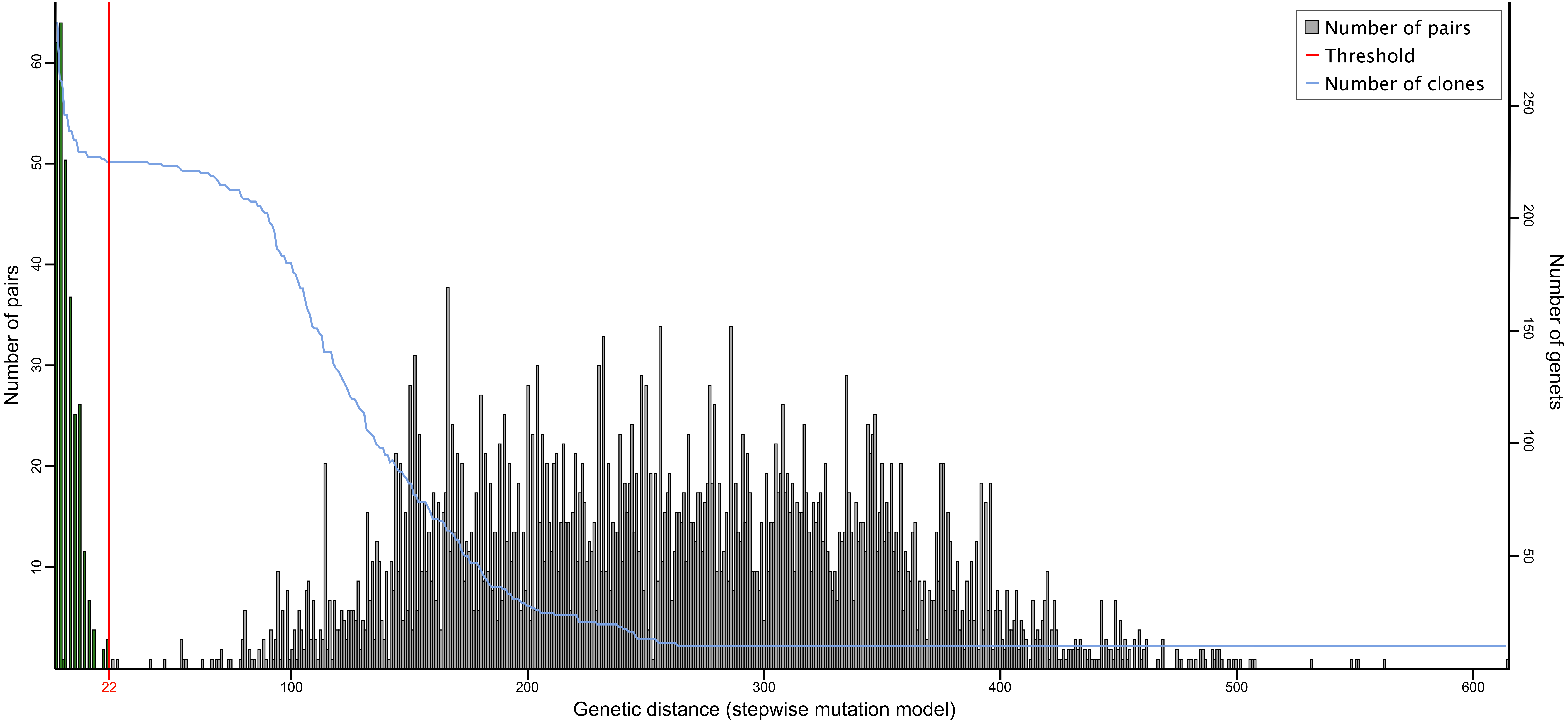

Supplementary Fig. S1. Distribution of pairwise genetic distances between all individuals, calculated under a stepwise mutation model. Genetic distance is defined as the minimum number of mutation steps required to transform one individual's genotype into another's. The histogram (gray bars) represents the number of pairwise comparisons at each genetic distance. The blue line shows the number of genets identified at every possible threshold, and the red line indicates the chosen threshold for defining distinct genets.

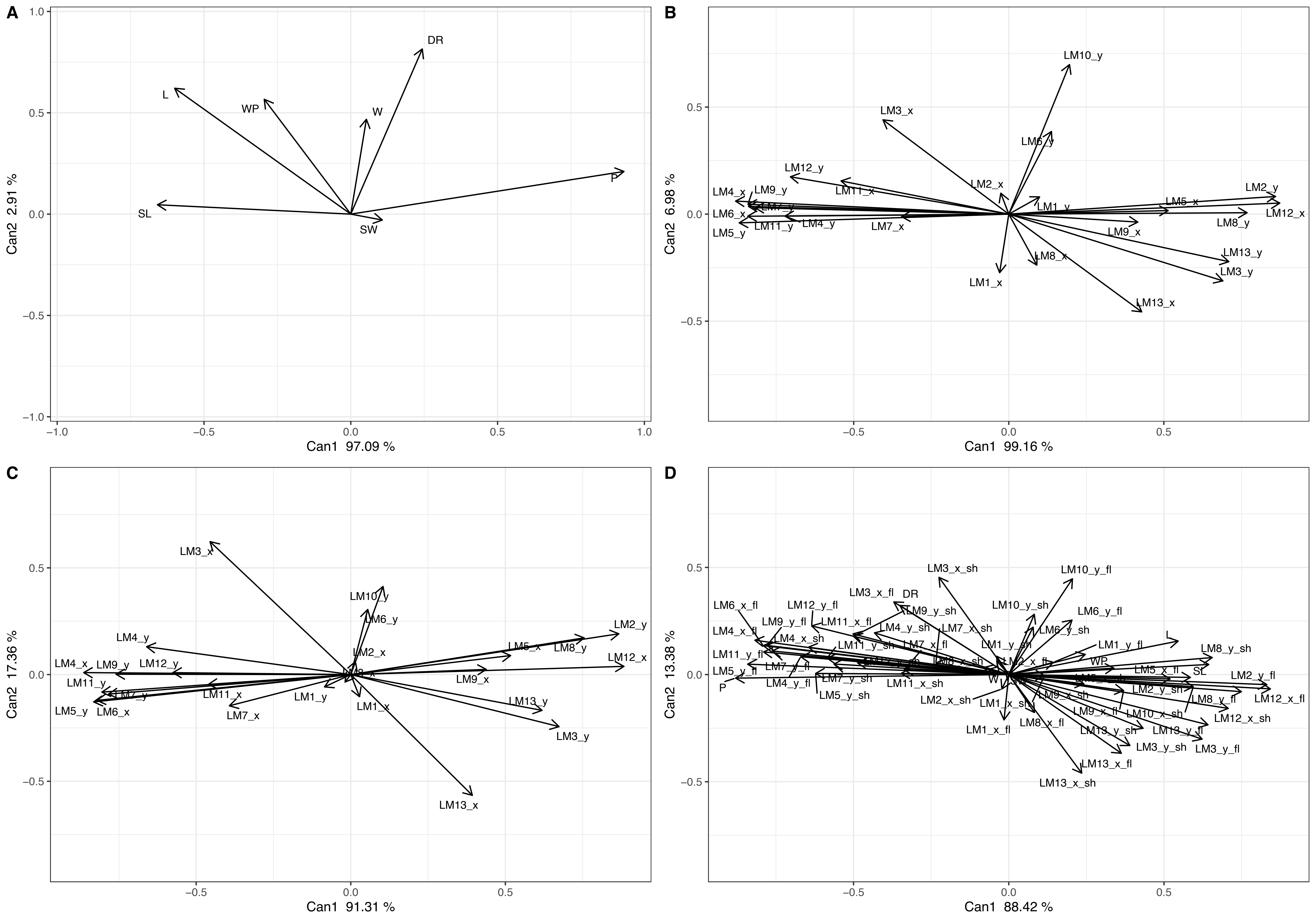

Supplementary Fig. S2. Loadings of morphological traits on the first two canonical variates (Can1 and Can2) from the Canonical Discriminant Analysis (CDA) shown in **Fig. 5**. The percentage of variance explained by Can1 and Can2 is indicated on each axis for each subplot. The loadings are presented for: **(A)** fruit traits, **(B)** leaves from flowering shoots, **(C)** leaves from short shoots, **(D)** the combined dataset. The length and direction of the vectors represent the contribution of each trait to the canonical axes, indicating their relative influence on species separation. For the abbreviation explanation, see the caption to **Fig. 1**.

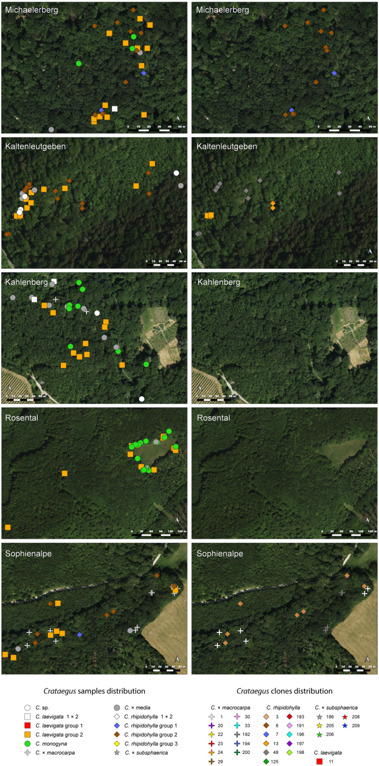

Supplementary Map S1. *Crataegus* samples and clones distribution in Austria

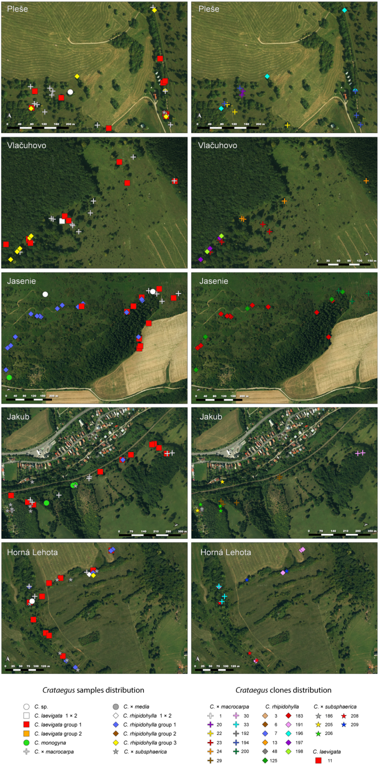

Supplementary Map S2. *Crataegus* samples and clones distribution in Slovakia
